# Adipokine networks in diabetic kidney disease: mechanistic insights and therapeutic implications

**DOI:** 10.1186/s12944-025-02851-9

**Published:** 2026-01-10

**Authors:** Ke Yang, Yuyang Fang, Junbo He, Jing Li

**Affiliations:** 1 Dongzhimen Hospital, Beijing University of Chinese Medicine, Beijing, China; 2 Beijing University of Chinese Medicine, Beijing, China

**Keywords:** Diabetic nephropathies, Adipokines, Lipid metabolism, Inflammation, Oxidative stress, Fibrosis, Systems biology, Precision medicine

## Abstract

Diabetic kidney disease (DKD), the predominant microvascular complication of diabetes mellitus, perpetuates a significant global health and socioeconomic challenge, complicating the pursuit of sustainable renal care. Adipokines, bioactive proteins secreted by adipose tissue that modulate lipid metabolism, function as key modulators potentially integrating systemic metabolic and inflammatory signals with renal pathophysiology Mechanistic investigations reveal that adipokines orchestrate a range of interconnected pathways, which include metabolic dysregulation (characterized by insulin resistance and lipid overload), immune-inflammatory responses (mediated by nuclear factor kappa B [NF-κB], NLR family pyrin domain containing 3 [NLRP3], and chemokine axes), oxidative stress coupled with mitochondrial dysfunction (involving adenosine monophosphate-activated protein kinase [AMPK] and peroxisome proliferator-activated receptor gamma coactivator 1-alpha [PGC-1α], reactive oxygen species [ROS]), endothelial dysfunction, fibrogenesis (driven by transforming growth factor beta [TGF-β]/Smad and epithelial-mesenchymal transition [EMT]), and the imbalance between apoptosis and autophagy. Protective adipokines such as adiponectin, irisin, and vaspin may mitigate harmful signaling, whereas leptin, resistin, visfatin, and chemerin could amplify injury through pro-inflammatory, pro-fibrotic, and lipotoxic pathways. Both circulating and urinary levels of adipokines may correlate with proteinuria, which suggests their potential utility in early detection, risk stratification, or therapeutic monitoring, although further validation is required.Emerging pharmacological, genetic, and lifestyle interventions may modulate adipokine networks to confer renal protection. The integration of multi-omics approaches, single-cell analysis, and spatial profiling with models that closely mimic human physiology is essential for identifying key signaling nodes, validating biomarkers, and developing precision-targeted therapies. Collectively, a detailed, network-oriented understanding of lipid-regulating adipokines could support efforts toward the development of personalized prevention and treatment strategies in DKD.

**Supplementary Information:**

The online version contains supplementary material available at 10.1186/s12944-025-02851-9.

## Introduction

Diabetes mellitus (DM) constitutes a globally prevalent chronic metabolic disorder, which is categorized into type 1 diabetes mellitus (T1DM) and type 2 diabetes mellitus (T2DM). Among its complications, diabetic kidney disease (DKD) emerges as one of the most common and severe, afflicting approximately 50% of individuals with T2DM and 33% of those with T1DM [1]. DKD has become the foremost cause of end-stage renal disease (ESRD) and significantly amplifies cardiovascular risk, thereby imposing a substantial clinical and socioeconomic burden. The Global Burden of Disease (GBD) study reports that the age-standardized incidence rate (ASIR) of DKD associated with T2DM increased by 21.0% between 1990 and 2021, whereas the ASIR for DKD due to T1DM saw a rise of 19.3% during the same timeframe [2, 3]. These figures are derived from complex modeling of diverse data sources, distinguishing between types of diabetes and adjusted for age structure, yet they continue to face limitations inherent in diagnostic coding and data availability across various regions. Such trends underscore the pressing need for innovative preventive and therapeutic strategies.

The escalating burden of DKD challenges the glucocentric perspective of its pathogenesis and unveils a complex interplay of metabolic, inflammatory, and fibrotic pathways [4]. Adipose tissue (AT), traditionally regarded merely as an energy storage depot, is now recognized as a dynamic endocrine organ. This paradigm shift began with the discovery of leptin in the 1990s [5]. As a crucial metabolic hub, AT secretes an array of bioactive proteins known as adipokines, which include adiponectin, leptin, resistin, and apelin. These molecules exert systemic effects on energy metabolism, insulin sensitivity, immune regulation, vascular homeostasis, redox balance, and fibrotic remodeling [6]. It is notable that adipokine expression and secretion can demonstrate sexual dimorphism and vary according to adipose depot, factors that may contribute to heterogeneity in DKD susceptibility and clinical presentation [7–9].

The current, often fragmented approach to studying individual adipokines does not sufficiently capture the complexity of their interactions in DKD. Therefore, a holistic, network-oriented perspective is critically needed to bridge the gap between isolated mechanistic insights and the multifactorial nature of the disease. This review aims to provide such a synthesis. While we comprehensively explore the expanded roles of adipokines across metabolic, inflammatory, and fibrotic axes, our primary goal is to promote a more integrated understanding that can facilitate the translation of adipokine biology into novel diagnostic and therapeutic strategies for DKD. Adopting this network-based framework, rather than focusing narrowly on a single pathway, is essential for decoding the systemic nature and heterogeneity of DKD, and represents the innovative aspect of this work.

## Classification and network of adipokines

AT serves as a metabolically active endocrine organ that secretes adipokines, which are crucial in regulating energy metabolism, insulin sensitivity, inflammation, and fibrosis via endocrine, autocrine, and paracrine mechanisms. Adipokines are broadly categorized into classical and emerging groups based on the timeline of their discovery and the extent of their characterization (Table 1).

To elucidate their synergistic effects in DKD, adipokines may be considered interconnected elements within a metabolic-inflammatory signaling network that impacts long-term renal outcomes.

**Table 1:** Overview of classical and emerging adipokines

Category	Adipokine	Source/Expression	Core Biological Features/Functions
Classical	Adiponectin	Adipocytes (subcutaneous WAT predominant)	Enhances insulin sensitivity; anti-inflammatory; anti-atherosclerotic
Classical	Leptin	Adipocytes; expression correlates with fat mass	Regulates appetite and energy expenditure; modulates mitochondrial activity; influences inflammation
Classical	Resistin	Macrophages (predominant in humans)	Links obesity to insulin resistance; pro-inflammatory cytokine induction
Classical	Apelin	Adipocytes, multiple organs	Activates AMPK; improves glucose/lipid metabolism; inhibits fibrosis
Emerging	Visfatin (NAMPT/PBEF)	Visceral AT, macrophages	NAD⁺ synthesis (intracellular); pro-inflammatory mediator (extracellular)
Emerging	Vaspin	Visceral AT	Anti-inflammatory; regulates ER stress; inhibits apoptosis
Emerging	Chemerin	Adipocytes, liver	Modulates immunity and metabolism; promotes ECM deposition
Emerging	Irisin	Skeletal muscle, AT	Induces browning of white fat; antioxidant; metabolic improvement
Emerging	Lipocalin-2 (LCN2/NGAL)	Multiple tissues (adipocytes, kidney)	Inflammation, iron metabolism; early biomarker of tubular injury

This table summarizes the classification of classical and emerging adipokines, as well as their primary sources and core biological functions

*WAT* White adipose tissue, *AMPK* AMP-activated protein kinase, *ECM* Extracellular matrix, *ER* *Stress* Endoplasmic reticulum stress, *NAD*⁺ Nicotinamide adenine dinucleotide

### **C**lassical adipokines

Classical adipokines, such as adiponectin, leptin, resistin, and apelin, are well recognized for their roles in metabolic regulation, inflammation, and the pathogenesis of DKD.

#### Adiponectin

Adiponectin is predominantly secreted by adipocytes and is characterized by its collagen-like domains and globular regions [10]. It exerts its effects through several receptors, including adiponectin receptor 1/2 (AdipoR1/R2), calreticulin, and T-cadherin [11]. The interaction between its globular domain and T-cadherin not only inhibits atherosclerosis [12] but also promotes exosome biogenesis and reduces ceramide synthesis [13], enhancing insulin sensitivity and mitigating insulin resistance [14, 15]. Collectively, these mechanisms contribute to metabolic and cardiovascular protection.

#### Leptin

Leptin, synthesized by adipocytes, is modulated by lipid accumulation, LEP gene expression, and cell size. In the hypothalamus, it diminishes the activity of neuropeptide Y (NPY) and gamma-aminobutyric acid (GABA) neurons, elevates corticotropin-releasing hormone (CRH) expression, curtails appetite, and augments energy expenditure [16]. Within adipocytes, leptin boosts mitochondrial activity and fatty acid oxidation [17], while concurrently inhibiting pancreatic insulin secretion [18]. In cases of obesity, leptin resistance compromises these functions, leading to hyperinsulinemia and weight gain. Furthermore, leptin activates nuclear factor kappa B (NF-κB), which promotes inflammation [5] and influences glomerular filtration, potentially facilitating early proteinuria in DKD.

#### Resistin

Initially identified as a mediator between obesity and insulin resistance, resistin, also known as found in inflammatory zone 3 (FIZZ3), is predominantly secreted by macrophages in humans. It activates NF-κB through Toll-like receptor 4 (TLR4), catalyzing the secretion of pro-inflammatory cytokines such as C-reactive protein (CRP), tumor necrosis factor-alpha (TNF-α), and interleukin-6 (IL-6) [19]. Transgenic mice overexpressing human resistin exhibit exacerbated AT inflammation, increased skeletal muscle lipid accumulation, and compromised insulin signaling under high-fat diet conditions [20].

#### Apelin

Apelin, an endogenous ligand for the APJ G protein-coupled receptor, is secreted by adipocytes and various other tissues. The apelin-APJ axis activates adenosine monophosphate-activated protein kinase (AMPK), inhibits the transforming growth factor-beta (TGF-β)/Smad signaling pathway to ameliorate renal interstitial fibrosis [21], and enhances glucose uptake and lipid metabolism through the AMPK, phosphoinositide 3-kinase/protein kinase B (PI3K/Akt), and endothelial nitric oxide synthase (eNOS) pathways [22]. A randomized double-blind trial has demonstrated that exogenous administration of apelin significantly enhances insulin sensitivity in overweight men [23].

##### Summary

Classical adipokines serve as pivotal regulators in the pathogenesis of DKD, orchestrating metabolic and inflammatory signals between AT and other organs. Their functions provide a foundational mechanism that allows newer adipokines to further diversify and enhance adipose-renal communication within the context of metabolic diseases.

### Emerging adipokines

Emerging adipokines augment the functions of classical adipokines by modulating metabolism, inflammation, and fibrosis, each assuming distinct and context-dependent roles in DKD.

#### Visfatin (nicotinamide phosphoribosyltransferase, NAMPT; pre-B cell colony-enhancing factor, PBEF)

Visfatin operates intracellularly as an NAD⁺ synthase (iNAMPT) and extracellularly as a pro-inflammatory cytokine (eNAMPT). The latter activates NF-κB via TLR4, inducing expression of IL-6 and TNF-α, and promoting endothelial dysfunction [24–29]. In DKD, elevated visfatin levels are associated with mesangial cell proliferation, markers of tubular fibrosis, severity of proteinuria, and reduced estimated glomerular filtration rate (eGFR) [30, 31].

#### Vaspin

Vaspin, a serine protease inhibitor, attenuates NF-κB signaling and diminishes the release of pro-inflammatory cytokines [32–34]. It also influences endoplasmic reticulum (ER) stress and lysosomal function, inhibits activation of the NLRP3 inflammasome, and reduces apoptosis in tubular epithelial cells [35].

#### Chemerin

Chemerin modulates metabolism and immunity via chemokine-like receptor 1 (CMKLR1) [36, 37]. In DKD, it activates pro-inflammatory p38 mitogen-activated protein kinase (MAPK) and NF-κB pathways and promotes extracellular matrix deposition and renal fibrosis through TGF-β1/Smad signaling [38–40].

#### Irisin

Secreted in response to exercise, irisin fosters the browning of WAT and enhances systemic metabolic function [41]. It activates sirtuin 1 (SIRT1)/nuclear factor erythroid 2-related factor 2 (Nrf2) signaling to mitigate oxidative stress [42], inhibits dynamin-related protein 1 (Drp1)-mediated mitochondrial fission, prevents vascular smooth muscle calcification, and has shown renoprotective effects in chronic kidney disease (CKD) models [43].

#### Lipocalin-2 (LCN2/neutrophil gelatinase-associated lipocalin, NGAL)

Lipocalin-2 is expressed in various tissues and regulates inflammation, iron metabolism, and energy homeostasis. It is an early biomarker of tubular injury [44, 45]. In DKD, elevated levels correlate with proteinuria and renal dysfunction [46], and it may have dual effects on fibrosis [47].

##### Summary

Both classical and emerging adipokines are integral to the metabolic, inflammatory, and fibrotic pathways. Their dysregulated expression in DKD underscores a complex endocrine-mediated network that links AT with kidney function. The subsequent section will delve into their mechanistic roles in the pathogenesis of DKD.

## Mechanistic roles of adipokines in diabetic kidney disease

**Fig. 1:**
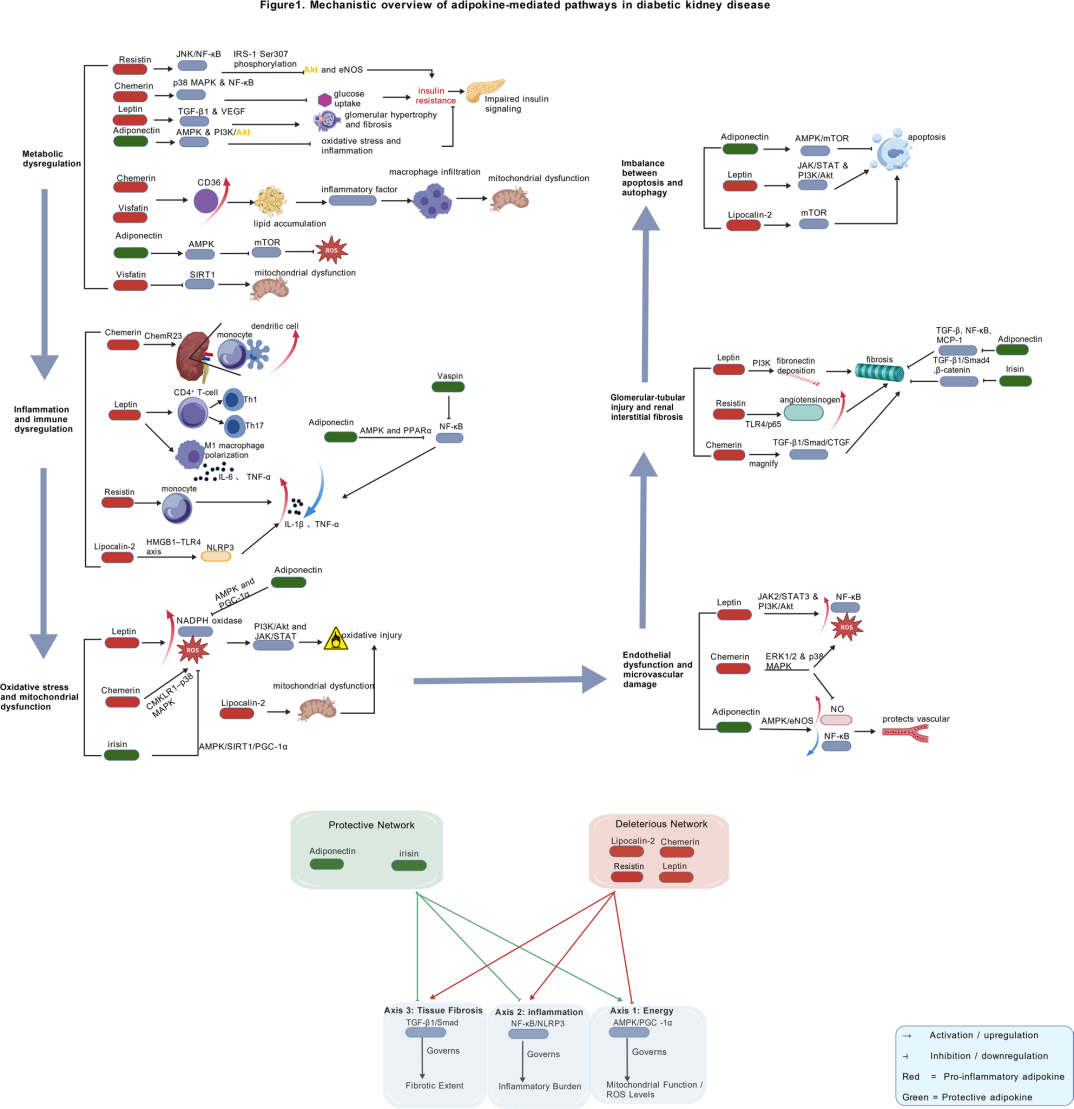
Mechanistic overview of adipokine-mediated pathways in DKD

Accumulating evidence suggests that adipokines play multifaceted roles that extend beyond energy regulation, directly influencing the pathogenesis of DKD. These adipokines do not act in isolation but function as interconnected components within a larger signaling network. This network compromises insulin signaling, activates inflammatory and inflammasome pathways, induces oxidative stress, disrupts the balance between apoptosis and autophagy, and promotes fibrosis and tissue remodeling. The subsequent sections elaborate on these mechanisms, emphasizing the crosstalk and integration that characterize the adipokine network as a whole, as well as its tissue-specific impacts on glomeruli, proximal tubules, and the renal microvasculature (refer to Table 2 and Fig. 1).

**Table 2:** Key adipokines and their dominant mechanistic effects in diabetic kidney disease (DKD)

Adipokine	Direction	Key Pathways	Principal Effects in DKD
Adiponectin	Protective	AMPK, PPARα, PI3K/Akt	↓Inflammation, ↓ROS, ↑mitochondrial function, anti-fibrotic
Leptin	Harmful	JAK/STAT, PI3K/Akt, TGF-β1	↑Fibrosis, ↑ROS, podocyte injury, glomerular hypertrophy
Chemerin	Harmful	CMKLR1, MAPK, NF-κB	↑Inflammation, ↑ROS, endothelial dysfunction, tubular injury
Lipocalin-2 (NGAL)	Harmful	TLR4, NLRP3, NADPH oxidase	Mitochondrial dysfunction, ↑fibrosis, ↑apoptosis
Resistin	Harmful	ER stress, NF-κB, JNK	↑Insulin resistance, ↑inflammation, promotes renal fibrosis
Irisin	Protective	AMPK/SIRT1/PGC-1α	↑Mitochondrial biogenesis, ↓ROS, anti-fibrotic, endothelial protection

This table summarizes key adipokines and their dominant mechanistic effects in DKD. For a more detailed and comprehensive list, see Supplementary Table 2

*Abbreviations*: *AMPK* Adenosine monophosphate-activated protein kinase, *PPAR*α Peroxisome proliferator-activated receptor alpha, *PI3K* Phosphoinositide 3-kinase, *Akt* Protein kinase B, *JAK/STAT* Janus kinase / signal transducer and activator of transcription, *TGF*-*β1* Transforming growth factor beta 1, *CMKLR1* Chemokine-like receptor 1, *MAPK* Mitogen-activated protein kinase, *NF*-*κB* Nuclear factor kappa B, *ROS* Reactive oxygen species, *NLRP3* NLR family pyrin domain containing 3, *NADPH* Nicotinamide adenine dinucleotide phosphate, *ER* Endoplasmic reticulum, *JNK* C-Jun N-terminal kinase, *SIRT1* Sirtuin 1, *PGC-1*α Peroxisome proliferator-activated receptor gamma coactivator 1-alpha, *NGAL* Neutrophil gelatinase-associated lipocalin

Arrows indicate direction of effect: ↑ increase;↓ decrease

The intricate interplay among adipokines is conceptually summarized through three core, antagonistic signaling axes that integrate their collective effects on renal pathology:

- The Energy & Oxidative Stress Axis (AMPK/PGC-1α), modulated by both protective (e.g., adiponectin) and deleterious (e.g., leptin) adipokines.
- The Inflammation & Immunity Axis (NF-κB/NLRP3), activated by pro-inflammatory adipokines and suppressed by protective ones.
- The Tissue Fibrosis Axis (TGF-β/Smad), driven by pro-fibrotic adipokines and inhibited by anti-fibrotic agents.
- The dynamic equilibrium between these opposing networks at shared signaling nodes ultimately dictates the renal outcomes in DKD. This figure was generated using BioGDP.com [48] (license number: GDP2025I2HEB2).

### Metabolic dysregulation

#### Impaired insulin signaling

Impaired insulin signaling constitutes an early and crucial mechanism in the pathogenesis of DKD. Multiple adipokines are implicated in this impairment. Resistin, for instance, induces ER stress and activates c-Jun N-terminal kinase (JNK) and NF-κB, leading to phosphorylation of IRS-1 at Ser307, which inhibits Akt and eNOS activity, thereby exacerbating insulin resistance in renal cells [49]. Chemerin activates p38 MAPK and NF-κB, reduces phosphorylation of IRS-1/Akt, impairs glucose uptake, and is associated with clinical manifestations of insulin resistance [50, 51]. Elevated leptin levels, often found in obesity and hyperinsulinemia, upregulate TGF-β1 and vascular endothelial growth factor (VEGF), which promote glomerular hypertrophy and fibrosis [52]. Conversely, adiponectin enhances insulin signaling through AMPK and PI3K/Akt pathways, mitigating oxidative stress and inflammation; its deficiency is associated with podocyte injury and proteinuria [53]. The ratio of leptin to adiponectin has been proposed as a marker of the metabolic-inflammatory status in DKD [54].

#### Lipid overload

Excessive lipid accumulation directly contributes to renal injury, extending beyond its impact on insulin resistance. Visfatin and chemerin upregulate the scavenger receptor CD36, which promotes lipid deposition, activates NF-κB, and increases expression of MCP-1 and TGF-β1. These changes lead to macrophage infiltration and fibrogenesis, often accompanied by mitochondrial dysfunction [55]. Clinically, elevated serum levels of visfatin are correlated with proteinuria and reduced renal function [56].

#### Energy sensing dysregulation

Dysregulation in energy sensing exacerbates metabolic injury significantly. The signaling pathway of adiponectin-AMPK enhances fatty acid β-oxidation, inhibits the activation of mTOR, reduces the production of reactive oxygen species (ROS), and preserves nephron integrity [57, 58]. In contrast, elevated levels of visfatin suppress SIRT1 activity, impair mitophagy, and disrupt energy homeostasis, thereby aggravating renal damage [59].

#### Integrated adipokine network and net effect

Adipokines function within a dynamic network that exhibits both opposing and synergistic effects. Adiponectin attenuates the detrimental impacts of resistin and leptin, while chemerin and visfatin intensify lipotoxic and glucotoxic stress. This interaction contributes to the clinical heterogeneity observed in DKD and may affect therapeutic responses, indicating that strategies should focus on restoring the balance of the entire network rather than targeting isolated factors.

### Inflammation and immune dysregulation

#### Central role of inflammation

A chronic, low-grade inflammatory state is a primary driver of DKD, with adipokines coordinating both local and systemic immune responses that exacerbate disease progression.

#### Immune cell recruitment and polarization

Chemerin, which is upregulated in the kidneys during DKD, promotes chemotaxis of monocytes and dendritic cells through ChemR23 [60]. Leptin facilitates the differentiation of CD4⁺ T-cells into pro-inflammatory Th1 and Th17 subtypes and promotes M1 macrophage polarization, thereby enhancing the production of IL-6 and TNF-α and linking metabolic disturbances to immune activation [61]. Resistin provokes peripheral monocytes to secrete IL-1β and TNF-α, and it enhances endothelial adhesion, thereby facilitating leukocyte recruitment to renal tissues [62].

#### Inflammasome and signaling pathway activation

Chemerin exacerbates tubular pro-inflammatory responses by activating the MAPK and NF-κB pathways, leading to increased cytokine production and localized inflammation [63]. Lipocalin-2 triggers the NLRP3 inflammasome via the HMGB1-TLR4 axis, inducing the maturation and release of IL-1β and IL-18. This mechanism contributes to renal fibrosis and underscores the crosstalk between inflammation and tissue remodeling mediated by adipokines [64, 65].

#### Protective adipokines

Adiponectin inhibits NF-κB activation via the AMPK and PPARα pathways, thereby reducing the production of TNF-α and IL-1β [52]. Vaspin mitigates ERK/NF-κB signaling in tubular cells, diminishing the expression of pro-inflammatory factors and counterbalancing the effects of pro-inflammatory adipokines [66].

#### Network regulation and metabolic interplay

Adipokines operate within a complex network, featuring feedback loops that integrate metabolic and inflammatory signals. For example, leptin induces TNF-α in a positive feedback loop, which is mitigated by adiponectin’s effects. Concurrently, the NF-κB-mediated inhibition of IRS-1/PI3K/Akt signaling exacerbates insulin resistance, creating a deleterious cycle central to the progression of DKD and influenced by the overall state of the adipokine network [67, 68].

### Oxidative stress and mitochondrial dysfunction

#### Excessive ROS and impaired antioxidant defense

Oxidative stress constitutes a fundamental pathogenic mechanism in DKD, significantly influenced by adipokines that contribute to renal injury. Under conditions of hyperglycemia, disruptions in glucose and lipid metabolism coincide with protein kinase C (PKC) activation, leading to a substantial increase in the production of ROS within renal cells [69, 70]. This increased mitochondrial workload in mesangial cells, podocytes, and tubular epithelial cells, accompanied by a simultaneous decline in antioxidant defenses, results in sustained oxidative damage.

#### Mitochondrial structural and functional impairment

Persistent oxidative stress inflicts direct damage on mitochondria, compromising their structural integrity and bioenergetic functionality. This damage is characterized by a reduced membrane potential, impaired ATP synthesis, mutations in mitochondrial DNA, and dysregulated autophagy [71, 72]. Importantly, these compromised mitochondria release mitochondrial damage-associated molecular patterns (mtDAMPs), which activate innate immune receptors such as TLRs and the NLRP3 inflammasome, thereby initiating a feed-forward loop that exacerbates inflammation and fibrosis [73].

#### Adipokine-mediated regulation of oxidative stress

Adipokines play critical and contrasting roles in regulating oxidative balance. Protective adiponectin activates the AMPK/PGC-1α pathway, enhancing mitochondrial biogenesis, inhibiting NADPH oxidase activity, and thus maintaining mitochondrial health [53, 74]. Conversely, leptin promotes ROS generation through NADPH oxidase and stimulates the PI3K/Akt and JAK/STAT pathways, intensifying oxidative damage [75–77]. Similarly, chemerin augments ROS production and inflammatory responses via its CMKLR1 receptor and downstream p38 MAPK signaling [38, 78], while lipocalin-2 further disrupts mitochondrial equilibrium [79]. In contrast, irisin mitigates oxidative stress by activating the AMPK/SIRT1/PGC-1α pathway, thereby enhancing mitochondrial function and reducing harmful ROS accumulation [80, 81].

#### Metabolic-inflammatory cross-talk via oxidative stress

Oxidative stress and inflammation interact synergistically, reinforcing each other and accelerating the progression of DKD. Pro-inflammatory adipokines, such as chemerin and resistin, increase NADPH oxidase activity, raising ROS levels which, in turn, activate the NF-κB pathway. This activation establishes a positive feedback loop that perpetuates inflammation. Concurrently, leptin not only enhances ROS production but also reduces cellular antioxidant capacity, thereby impeding ROS clearance and further exacerbating renal damage [76, 82].

### Endothelial dysfunction and microvascular damage

#### Pathophysiological basis

Endothelial dysfunction is a principal factor driving the progression of DKD. Glomerular endothelial cells (GECs) are critical in maintaining vascular tone and the integrity of the filtration barrier. In diabetes, the combined effects of hyperglycemia, oxidative stress, and chronic inflammation collectively diminish nitric oxide (NO) availability, increase vascular permeability, and induce endothelial damage, which ultimately leads to microvascular lesions [83].

#### Endothelial activation and structural remodeling

Endothelial dysfunction is manifested by impaired eNOS activity under conditions of hyperglycemia and insulin resistance, which decreases NO synthesis. Elevated levels of adhesion molecules, such as ICAM-1 and VCAM-1, facilitate the adhesion and transmigration of leukocytes. Persistent endothelial activation leads to apoptosis and structural alterations, including thickening of the basement membrane [84, 85].

#### Adipokine-mediated regulation of endothelial function

Adipokines exhibit dichotomous effects on endothelial homeostasis.

Protective adipokines, such as adiponectin and irisin, enhance NO availability through AMPK-dependent eNOS activation and inhibit NF-κB signaling, thereby preserving vascular function [86–88].

Conversely, pro-inflammatory adipokines contribute to endothelial dysfunction: leptin upregulates adhesion molecules through the JAK2/STAT3 and PI3K/Akt pathways; chemerin enhances ROS production and inflammatory signaling through ERK1/2 and p38 MAPK; and lipocalin-2 impairs mitochondrial function and increases vascular permeability [36, 89–92].

#### Consequences of endothelial injury

Persistent endothelial damage accelerates the formation of microvascular lesions, leading to renal ischemia and hypoxia. This, in turn, activates the hypoxia-inducible factor 1-alpha (HIF-1α) and TGF-β pathways. While pro-inflammatory adipokines exacerbate this cycle of injury, protective adipokines counteract these effects and maintain vascular integrity, thus critically influencing the progression of DKD [93].

### Glomerular-tubular injury and renal interstitial fibrosis

#### Pathological progression

In DKD, injury to the glomeruli and proximal tubules is synergistic and mutually reinforcing, which accelerates renal interstitial fibrosis and functional decline. Hyperglycemia induces mesangial matrix expansion, capillary loop disorganization, and disruption of the podocyte slit diaphragm, collectively compromising the filtration barrier [94]. Concurrently, proximal tubular epithelial cells (PTECs), highly susceptible to glucotoxicity and proteinuric stress, exhibit mitochondrial dysfunction, lysosomal stress, and ferroptosis, further exacerbating renal injury [95, 96].

#### Fibrogenic mechanisms

Renal interstitial fibrosis is propelled by multiple convergent pathways, including the epithelial-to-mesenchymal transition (EMT) of PTECs, mesangial cell activation, and excessive extracellular matrix (ECM) deposition. Transforming growth factor-beta1 (TGF-β1) plays a pivotal role, activating Smad2/3 signaling to promote the expression of pro-fibrotic genes. Hypoxia further enhances TGF-β signaling through upregulation of HIF-1α, establishing a persistent positive feedback loop that perpetuates fibrosis [97].

#### Adipokine-mediated regulation of fibrosis

Adipokines significantly influence fibrogenic pathways, displaying both pathogenic and protective roles.

Pro-fibrotic adipokines include leptin, which upregulates the TGF-β receptor II (TβRII) in mesangial cells through PI3K signaling, promoting collagen and fibronectin deposition [98]; resistin, which activates TLR4/p65 signaling, enhancing angiotensinogen production and thereby augmenting RAS activation and inflammation [99, 100]; chemerin, which intensifies TGF-β1/Smad/CTGF signaling, while receptor antagonism reduces collagen accumulation [39]; and lipocalin-2, which exacerbates mitochondrial injury, ROS production, and apoptosis, worsening fibrosis [79].

Anti-fibrotic adipokines include adiponectin, which inhibits TGF-β, NF-κB, and MCP-1 pathways, delaying fibrosis and preserving renal architecture [101]; and irisin, which suppresses TGF-β1/Smad4 and β-catenin signaling, reverses EMT, and mitigates interstitial fibrosis [102].

#### Integrated effects and therapeutic implications

Collectively, adipokines form a complex regulatory network that modulates glomerular-tubular injury and interstitial fibrosis through overlapping and opposing signaling axes. Their dual roles, pathogenic and protective, therefore provide a mechanistic framework for understanding the heterogeneity of DKD and highlight promising pathways for novel therapeutic strategies.

### Imbalance between apoptosis and autophagy

#### Pathophysiological basis

In DKD, renal cells endure persistent metabolic insults due to hyperglycemia, oxidative stress, and chronic inflammation. This hostile microenvironment disrupts cellular homeostasis, resulting in a pathological imbalance between cell death and survival mechanisms. A significant increase in apoptotic cell death occurs simultaneously with the suppression of the protective autophagy pathway, collectively exacerbating structural damage and the loss of functional nephrons [103, 104].

#### Key dysregulated pathways

Apoptosis Activation: In DKD, the intrinsic (mitochondrial) apoptotic pathway predominantly mediates cell death. Mitochondrial outer membrane permeabilization is induced by ROS and glucose toxicity, characterized by the upregulation of Bax and the downregulation of Bcl-2, which leads to the activation of the caspase cascade and programmed cell death. Furthermore, ER stress, triggered by glucotoxicity, intensifies tubular apoptosis [103, 104].

Autophagy Suppression: Under diabetic conditions, the essential cellular clearance mechanism of autophagy is compromised. This impairment is evidenced by reduced levels of key autophagy markers such as Beclin-1 and LC3-II, alongside increased activity of the mechanistic target of rapamycin (mTOR). The accumulation of damaged mitochondria, protein aggregates, and other deleterious cellular components not only impairs cell function but also intensifies apoptotic signaling, perpetuating a cycle of injury [105].

#### Adipokine-mediated regulation of cell fate

Adipokines are critical extracellular signals that regulate the balance between apoptosis and autophagy within the diabetic kidney.

Protective Adipokines: Adiponectin activates AMPK signaling, which subsequently inhibits mTOR activity. This dual action enhances autophagic flux and diminishes ROS-induced apoptosis, thereby offering protection to renal cells [53].

Deleterious Adipokines: Leptin promotes apoptosis in podocytes and contributes to mesangial cell hypertrophy through the activation of JAK/STAT and PI3K/Akt signaling pathways [106]. Lipocalin-2 increases mTOR activity and induces DRP1-dependent mitochondrial fragmentation, effects that can be counteracted by the pharmacological inhibition of mTOR with rapamycin [79].

#### Integrated effects and pathological implications

The interactions among adipokines form a regulatory network that decisively influences the fate of renal cells. The predominance of pro-apoptotic signals (e.g., from leptin and lipocalin-2) over pro-survival and pro-autophagy signals (e.g., from adiponectin) drives nephron injury in DKD. This disruption of cellular homeostasis not only leads directly to cell loss but also contributes to the progression of renal dysfunction. Consequently, therapeutic strategies aimed at restoring this balance, either by inhibiting apoptosis or inducing protective autophagy, offer a promising approach to preserving renal structure and slowing disease progression.

## Controversies and divergences

Adipokines are recognized as pivotal regulators in DKD; however, significant inconsistencies concerning their expression, mechanisms, and clinical relevance impede their utilization as biomarkers or therapeutic agents. The primary sources of these divergences include interspecies variations, stages of disease progression, cellular environments, and comorbid conditions in patients.

### Heterogeneity of expression and clinical associations

The clinical implications of many adipokines are known to be context-sensitive. Visfatin, for instance, has been identified as elevated and associated with proteinuria in certain patient cohorts [56], yet it exhibits weak or no correlation with the decline in eGFR in other groups, where it is instead linked to systemic inflammation [107]. Preclinical studies occasionally propose protective roles for visfatin [108], adding complexity to its interpretation. Similar ambiguities are noted with chemerin and vaspin. Extrinsic metabolic factors, such as acute hyperbilirubinemia, which is implicated in early tubular injury even in mild conditions, may independently influence renal damage or adipokine signaling. This underscores the intricate pathophysiological context within which adipokines function [109], necessitating meticulous control for these variables to ensure accurate clinical interpretation.

### Mechanistic inconsistencies, tissue specificity, and translational insights

Adipokines demonstrate tissue- and context-dependent effects. For example, adiponectin predominantly exerts anti-inflammatory and antifibrotic actions in tubular cells through AMPK/PPARα-mediated inhibition of TGF-β [110]. However, under lipopolysaccharide (LPS) stimulation, adiponectin can induce pro-inflammatory responses in HK-2 cells [111]. Chemerin facilitates the recruitment of dendritic and T cells [112], while vaspin reduces inflammation via modulation of ER stress [66]. Variations in cell type, temporal dynamics, and experimental conditions contribute to significant discrepancies in results.

Emerging technologies, such as single-cell and spatial transcriptomics, enable the mapping of adipokine receptor expression across diverse renal cell types, including podocytes, mesangial cells, and tubular cells. Integrating these spatially resolved datasets with multi-omics approaches (including transcriptomics, proteomics, and metabolomics) throughout different stages of disease facilitates the identification of crucial regulatory nodes that dictate biological outcomes. Such insights are instrumental in refining patient stratification and developing biomarker-driven therapeutic strategies, such as distinguishing between tubular injury-dominant and inflammation-predominant DKD for targeted adipokine signaling modulation.

### Limitations of preclinical models

The majority of mechanistic insights are derived from rodent models of obesity and diabetes, such as db/db and ob/ob mice, which exhibit metabolic and renal pathologies distinct from human DKD. Species-specific variations in adipokine expression, processing, and splicing, exemplified by irisin, pose additional challenges to translational efforts [113, 114]. Human-relevant experimental platforms, including kidney organoids, precision-cut tissue slices, and ex vivo human samples, are crucial for the validation of mechanisms and therapeutic targets.

### Neglected network complexity

Most research studies assess adipokines in isolation, neglecting the complex regulatory networks that govern their collective effects. In reality, adipokines often function synergistically or antagonistically through shared signaling pathways such as PI3K/Akt, AMPK, and SIRT1, as well as through the cross-regulation of inflammatory and fibrotic mediators [115–117]. For example, leptin promotes the secretion of TNF-α and IL-6, which in turn influence the expression of chemerin, while adiponectin and visfatin modulate the PI3K/Akt and ER stress pathways, respectively. Overlooking these interactions can lead to oversimplified interpretations of mechanisms and diminish translational relevance. To systematically illustrate these context-dependent effects and address the ongoing debates, we summarize the key controversies and future research priorities for major adipokines in Table 3.

Integrating network-oriented analyses with multi-omics datasets facilitates the identification of central regulatory hubs and aids in the prioritization of combination interventions. Models that are relevant to human physiology, such as kidney organoids or precision-cut tissue slices, can validate these network-level hypotheses under the pathophysiological conditions of DKD.

**Table 3:** Sources of controversy and proposed resolution pathways for selected adipokines in DKD

Adipokine	Reported Effect in DKD	Species/Model	Experimental Context	Net Outcome	Notes / Future Research
Adiponectin	Anti-inflammatory, anti-fibrotic	Human tubular cells	Baseline conditions	Protective	Under LPS stimulation: pro-inflammatory; future: integrate human biopsy + organoid studies; stratify patients by receptor expression
Chemerin	Pro-inflammatory, promotes dendritic/T-cell recruitment	Rodent DKD models	Variable	Context-dependent	Resolve via multi-omics, cell-type-specific and spatial transcriptomics; patient stratification
Vaspin	Anti-inflammatory, ER stress modulation	Rodent DKD models	Variable	Protective	Assess human tubular and mesangial cells; consider receptor profiling; integrate organoid validation
Leptin	Induces TNF-α/IL-6, pro-fibrotic	Rodents & humans	Chronic hyperglycemia	Detrimental	Explore combination therapy with anti-inflammatory modulators; validate network interactions
Visfatin	Variable; sometimes pro-inflammatory, sometimes protective	Human cohorts, db/db mice	Obesity, inflammation	Context-dependent	Stratify by patient phenotype; integrate urinary/serum biomarker profiling; organoid and biopsy validation
Irisin	Renoprotective in preliminary studies	Rodent DKD models	Exercise / pharmacological induction	Protective	Validate in human organoids and biopsy tissue

Future research priorities focus on leveraging human-relevant models (e.g., organoids, precision-cut tissue slices) and integrating multi-omics to elucidate context-specific effects and enable patient stratification

*Abbreviations*: *DKD* Diabetic kidney disease, *LPS* Lipopolysaccharide

### Gaps in clinical application and standardization

Despite extensive mechanistic insights, the clinical application of adipokines in DKD remains limited by major gaps in measurement standardization and biomarker qualification. Currently, no consensus exists on reference ranges, diagnostic thresholds, or risk-stratification cutoffs, largely due to assay heterogeneity and insufficient cross-cohort reproducibility. Large-scale, multi-center cohorts employing harmonized protocols are required to establish clinically meaningful ranges and evaluate prognostic utility.

In addition, the regulatory pathway for incorporating adipokines into clinical decision-making remains poorly defined. Standardized reporting frameworks, inter-laboratory calibration, and longitudinal validation are needed before adipokines can be integrated into diagnostic algorithms or therapeutic monitoring. These unmet needs—rather than pharmacological feasibility—constitute the primary barriers to clinical translation at present.

### Sex and body composition as sources of heterogeneity

In recognition of substantial biological heterogeneity among individuals with DKD, sexual dimorphism and adipose tissue distribution emerge as important, yet often under‑appreciated determinants of adipokine biology and renal disease risk.

Evidence from large population‑based cohorts shows that, even after adjustment for total or visceral adiposity, females generally have higher circulating adiponectin and leptin concentrations compared to males [118]. This sex difference in adipokine levels cannot be fully explained by fat mass or fat distribution, suggesting additional sex‑specific regulatory mechanisms [119].

Studies indicate that sex hormones play a critical role: in human adipocytes, exposure to male versus female serum differently affects adipokine regulation. While direct effects of sex steroids on adiponectin secretion remain equivocal, the observed sexual dimorphism in circulating adipokines likely reflects complex endocrine regulation mediated by sex hormones in vivo [120].

Moreover, adipose tissue depot (e.g., subcutaneous vs. visceral) displays distinct adipokine secretion capacities, and these differences show evidence of sex‑dependence. For instance, subcutaneous adipose tissue (SAT) from humans releases more adiponectin than paired visceral adipose tissue (VAT) in in vitro incubation systems [121]. In addition, in a cohort study of African American men and women, VAT was inversely associated with serum adiponectin in women, but not in men — while in men, SAT showed a positive association with adiponectin [122].

Mechanistically, these sex- and depot-specific adipokine profiles may influence renal vulnerability in DKD. Individuals with relatively high adiponectin levels, which are more commonly observed in females or in those with favorable fat distribution such as greater subcutaneous adipose tissue, are likely to activate protective signaling pathways, including AMPK, PI3K/Akt, anti-inflammatory, antioxidant, and antifibrotic mechanisms, thereby potentially mitigating DKD progression. Conversely, a profile characterized by higher leptin or other pro-inflammatory adipokines combined with lower adiponectin, more prevalent in males or in individuals with central or visceral adiposity, may predispose to pro-inflammatory, lipotoxic, and profibrotic renal injury.

## Therapeutic implications

The adipokine network serves as a dynamic interface between metabolism and renal pathology, presenting both a source of biomarkers and a platform for therapeutic intervention in DKD. However, transitioning from mechanistic insights to clinical applications requires a precision medicine approach. This chapter details how employing network-oriented and multi-omics strategies can bridge this gap, facilitating risk stratification, targeted therapies, and ultimately, enhanced renal outcomes. 

### Risk assessment and early detection

Circulating and urinary adipokine levels serve as reflections of DKD severity and progression, offering potential biomarkers for early risk stratification. Elevated levels of chemerin and leptin are associated with systemic low-grade inflammation and are predictive of a decline in glomerular filtration rates [123]. Conversely, urinary adiponectin acts as an early marker of renal injury and responsiveness to treatment [57]. The incorporation of these adipokines into multi-marker panels, possibly integrated with transcriptomic or proteomic profiles, could enhance both the sensitivity and specificity of early detection methods. From a mechanistic standpoint, these biomarkers encapsulate the cumulative effects of metabolic dysregulation, inflammation, and fibrotic signaling, thereby facilitating a comprehensive assessment of DKD risk. Multi-omics approaches and network analyses further support the identification of patient subgroups who might benefit from tailored interventions.

### Therapeutic targeting of adipokines

Preclinical studies demonstrate that the direct modulation of adipokine signaling has the potential to mitigate DKD pathology. The administration of recombinant adiponectin or its agonists has been shown to activate AMPK and PI3K/Akt signaling pathways, which in turn reduce tubular oxidative stress, inflammation, and fibrosis [124]. Inhibition of chemerin decreases inflammation in glomerular endothelial cells and reduces extracellular matrix deposition [38]. Preliminary investigations into visfatin inhibitors and irisin analogs indicate renoprotective effects [108, 125]. These therapeutic interventions exemplify precision targeting of adipokine networks, providing mechanistically informed strategies for treatment. Key candidates, their mechanisms of action, and approaches for patient stratification aligned with these strategies are synthesized in Table 4.

**Table 4:** Strategies for targeting adipokine networks in DKD

Category	Intervention	Targeted Adipokines	Regulated Pathways / Mechanisms	Renal Effects	Precision Patient Stratification
Established & Repurposed Therapies	GLP-1R Agonists (GLP-1RA)	↑Adiponectin, ↓Leptin, ↓Resistin	AMPK, PI3K/Akt, anti-inflammatory signaling	Reduce tubular oxidative stress, inflammation, fibrosis	Patients with low adiponectin/high leptin; early or obesity-related DKD [125–126]
	SGLT2 Inhibitors (SGLT2i)	↓Pro-inflammatory adipokines	AMPK, metabolic homeostasis, oxidative stress pathways	Promote renal metabolic homeostasis; reduce inflammation	T2DM patients with DKD; early metabolic dysregulation (multi-omics identified) [128]
Lifestyle & Adjunctive Strategies	Lifestyle Interventions (exercise, weight loss, Mediterranean diet)	↑Adiponectin, ↓Leptin, ↓Chemerin	AMPK, SIRT1, anti-inflammatory & anti-fibrotic pathways	Improve metabolic-immune homeostasis; reduce systemic inflammation	Early-stage DKD, overweight/obese patients; stratified by baseline adipokine profile [127–129]
Emerging Biologics & Targeted Therapies	Adiponectin Agonists / Analogs	↑Adiponectin	AMPK, PI3K/Akt	Reduce oxidative stress, inflammation, fibrosis	High-risk DKD patients, multi-omics-guided selection [123]
	Chemerin Inhibition	Chemerin	NF-κB, endothelial activation	Reduces glomerular inflammation, ECM deposition	Patient subgroups with strong inflammatory signatures [38]
	Leptin Blockade	Leptin	TNF-α/IL-6, pro-fibrotic signaling	Attenuates glomerular injury	Patients with confirmed hyperleptinemia; combination therapy [89, 90]
	Irisin Analog Induction	Irisin	AMPK, mitochondrial function	Improves renal metabolism and fibrosis	Validate in pilot studies; patients with mitochondrial dysfunction [80, 81, 102]
	Vaspin Modulation	Vaspin	ER stress, anti-inflammatory	Attenuates tubular/mesangial inflammation	Receptor profiling; combined multi-omics studies [66]
	Visfatin Modulation	Visfatin	NAD+-dependent pathways, inflammatory modulation	Context-dependent (protective vs. pro-inflammatory)	Stratify by patient phenotype & biomarker panels [108, 124]
Next-Generation Platforms	Nanoparticle-based Delivery / Organoid-guided Therapy	Specific adipokines	Targeted delivery, enhanced bioavailability	Improve renal targeting and therapeutic efficacy	Advanced DKD; poor drug distribution; refined by single-cell/spatial transcriptomics

Integration with multi-omics techniques, encompassing transcriptomics, proteomics, and metabolomics, is crucial for identifying patient subgroups most likely to benefit from these therapeutic interventions. Single-cell and spatial transcriptomics could further elucidate the distribution of adipokine receptors, enhancing the precision of targeting. The potential for combination therapies to yield synergistic effects should be explored. To achieve clinical validation, large-scale prospective studies are indispensable

### Adjunctive strategies with established treatments

The current management of DKD predominantly employs renin-angiotensin system (RAS) blockers, sodium-glucose cotransporter 2 inhibitors (SGLT2is), and glucagon-like peptide-1 receptor agonists (GLP-1RAs). Although these interventions prove effective, a residual risk remains in certain patient subgroups. GLP-1RAs enhance glycemic control and alter adipokine profiles by increasing circulating adiponectin levels and decreasing leptin and resistin levels, which may attenuate renal inflammation, oxidative stress, and fibrosis [126, 127]. Similarly, SGLT2is have demonstrated an ability to raise adiponectin and reduce leptin levels, suggesting their indirect renoprotective effects stem from improved metabolic and inflammatory homeostasis [128]. These established therapies serve as broad network modulators, restoring balance to the dysregulated adipokine signaling landscape rather than targeting a single molecule. This broad modulation supports the rationale for patient stratification based on adipokine network signatures. For example, individuals presenting a ‘high-leptin/resistin, low-adiponectin’ profile, indicative of a pronounced metabolic-inflammatory drive, may particularly benefit from the adipokine-modulating effects of GLP-1RAs or SGLT2is.

Integration of multi-omics and network-oriented analyses assists in identifying patient subgroups likely to benefit most from these therapies. For instance, individuals characterized by low adiponectin/high leptin levels or elevated chemerin, as identified through transcriptomic or proteomic profiling, may respond preferentially to a combination of GLP-1RA and lifestyle interventions. Lifestyle modifications, such as structured exercise, weight reduction, and adherence to a Mediterranean-style diet, further modulate adipokine networks, enhance metabolic-immune balance, and support early-stage DKD management [129–131]. Coordinated pharmacologic and lifestyle strategies provide a practical approach to translate mechanistic insights into precision medicine applications, facilitating biomarker-guided, individualized therapy.

### Challenges and future directions

Building upon the clinical standardization gaps summarized in Sect. 4.5, this section focuses on mechanistic and technological avenues that may help overcome these translational barriers.

Despite promising preclinical and early clinical results, therapies targeting adipokines face significant challenges, including tissue specificity, rapid degradation or poor bioavailability of peptides, and incomplete characterization of downstream signaling pathways. Targeting stable and druggable nodes such as AMPK, SIRT1, or phosphoinositide 3-kinase/protein kinase B (PI3K/Akt) may alleviate some of these limitations. The use of nanoparticle-mediated delivery, organoid, or kidney-on-a-chip platforms could enhance mechanistic validation and tissue-specific targeting, facilitating rational therapeutic design.

Future research should incorporate human kidney biopsy–based multi-omics alongside in vitro and ex vivo models to refine adipokine-targeted approaches. Single-cell and spatial transcriptomics could map the distribution of adipokine receptors across renal cell types such as podocytes, mesangial cells, and tubular epithelial cells, enabling patient stratification and predictive biomarker-guided therapy. Patients could be stratified not only by clinical phenotype but also by molecular signatures, such as a “high-leptin/resistin, low-adiponectin” profile indicative of a strong metabolic-inflammatory drive, to prioritize them for specific adjunctive or targeted therapies.

Moreover, integrating biological variables such as sex and body composition into these models is critical, given their established impact on adipokine biology [7, 8]. For example, the more favorable adipokine profile observed in premenopausal females may influence disease progression and therapeutic responses, indicating that sex-specific treatment thresholds or personalized strategies may be necessary [7].

### Translation into clinical practice

To advance adipokine-based interventions, extensive multicenter, prospective studies are essential. These studies should encompass a variety of DKD subtypes, stages, and patient demographics, and must be adequately powered to facilitate sex-specific analyses. It is crucial that such studies routinely collect data on body composition and fat distribution, in addition to measuring circulating adipokine levels. The incorporation of adipokines into multimarker predictive models holds the potential to enhance risk stratification, improve the early detection of disease, and monitor therapeutic responses more effectively. For instance, integrating clinical data with adipokine profiles and molecular signatures derived from transcriptomics may facilitate the classification of patients into specific subtypes, such as ‘tubular-injury-predominant’ and ‘inflammatory-signature’ DKD. This classification could guide the selection of therapies that either target adipokine-mediated inflammation or address metabolic dysregulation. When combined with mechanistic validation from human-relevant platforms and network-level analyses, these strategies could underpin a new paradigm of precision medicine in DKD, moving away from a generic approach to one that is predictive, preventative, and personalized.

#### Summary

This review has thoroughly explored the significant roles of adipokines as central mediators and modulators in the pathogenesis of DKD. We have detailed the progression from dysregulated metabolic signaling and inflammatory activation to oxidative stress, endothelial dysfunction, and fibrosis, demonstrating how adipokine networks synthesize these fundamental pathological axes. Despite existing controversies regarding the context-specific actions of adipokines, the adoption of network-oriented and multi-omics approaches offers a definitive route forward. These methodologies aim to resolve existing conflicts and identify key regulatory hubs. The translation of this mechanistic understanding into clinical practice is now progressing, advancing through risk stratification with biomarker panels and the repurposing of existing metabolic therapies towards pioneering biologics and precision-targeted interventions. The future of DKD management will likely rely on embracing this complexity, transitioning from a uniform treatment strategy to a dynamic, network-informed model of precision medicine that holds promise for preserving renal function and enhancing patient outcomes.

## Strengths and limitations

### Strengths

This review offers a structured and integrative perspective on adipokine biology in DKD by bringing together findings from metabolism, inflammation, oxidative stress, and inter-organ communication. Rather than examining each adipokine in isolation, the review emphasizes their interconnections and outlines how alterations across this network may contribute to renal injury. This approach provides a coherent framework for understanding how multiple pathways converge in DKD and may help clarify potential points for earlier identification of risk or therapeutic intervention.

Another strength lies in its translational orientation. By linking mechanistic observations to current and emerging therapeutic strategies—including pharmacological agents, biologically active modulators, and technologies aimed at targeted delivery—the review highlights areas in which adipokine biology may eventually inform more individualized approaches to DKD management. The incorporation of systems biology, multi-omics analyses, and human-relevant models reflects ongoing trends in the field and may help contextualize how future research could refine biomarker development or improve treatment stratification.

Together, these elements aim to provide a balanced and organized overview of a rapidly evolving research area, summarizing current knowledge while outlining potential directions for further study.

### Limitations

Despite its breadth, this review has several important limitations. Much of the mechanistic evidence available in the literature originates from preclinical models, including rodent studies and in vitro systems, which may not fully reproduce the complexity of human DKD. As a result, many associations between adipokines and renal injury remain suggestive rather than causal, underscoring the need for longitudinal and mechanistic studies in diverse patient populations.

Variability in adipokine measurement further limits the comparability of published findings. Differences in assay platforms, calibration standards, and sample handling procedures often result in inconsistent reported concentrations, making it difficult to establish clinically applicable reference values. This heterogeneity complicates efforts to evaluate adipokines as reliable biomarkers for diagnosis, prognosis, or therapeutic monitoring.

In addition, population-based studies frequently involve small sample sizes, single-center recruitment, and limited ethnic representation. These constraints reduce the generalizability of adipokine-related observations and may obscure subgroup-specific patterns across sex, age, metabolic status, or disease stage. Cross-sectional study designs also restrict the ability to determine temporal changes in adipokine signaling over the course of DKD progression.

Although several therapeutic strategies targeting adipokine pathways have shown promise in experimental settings, translation to clinical practice is still in an early phase. Challenges related to delivery efficiency, tissue specificity, off-target effects, and long-term safety will need to be addressed before such interventions can be considered in routine care.

Taken together, these limitations highlight that the mechanistic and translational insights presented here should be interpreted with caution. Strengthening the evidence base will require standardized biomarker assays, multicenter longitudinal cohorts, and human-relevant experimental platforms—such as organoids and spatial multi-omics—to clarify the clinical relevance of adipokine signaling in DKD.

## Conclusion

Adipokines serve as crucial molecular intermediaries between metabolic dysfunction and renal injury in DKD. This review synthesizes evidence from metabolic, inflammatory, and fibrotic pathways, offering a network-based framework that may connect basic discoveries with potential clinical applications.

Mechanistic insights into adipokine signaling elucidate how imbalances within this network contribute to glomerular, tubular, and interstitial injury. Understanding these interactions may facilitate the early identification of high-risk individuals and enhances opportunities for biomarker-guided diagnosis, risk stratification, and therapeutic monitoring.

These insights may have clinical relevance, as they could help inform future efforts to translate molecular findings into decision-making tools for the management of DKD. Clinically, adipokine profiles could complement existing diagnostic panels and aid in tailoring treatment decisions, for example, guiding the selection or combination of SGLT2 inhibitors, GLP-1 receptor agonists, or other metabolic agents in particular patient subgroups. Targeting maladaptive adipokine signaling may help improve renal protection and potentially delay the progression of DKD beyond glucose control alone.

From a translational perspective, the integration of multi-omics platforms, organoid systems, and spatial profiling will likely expedite the validation of actionable adipokine targets. These efforts are consistent with the overarching aims of precision medicine, which seeks to convert mechanistic understanding into personalized care and sustainable health outcomes for the kidneys.

In summary, by linking molecular mechanisms with patient-centered strategies, adipokine research provides informative insights and may have potential clinical relevance. The mechanistic and network-oriented understanding of lipid-regulating adipokines presented here offers a framework that could support early detection, precision-guided interventions, and long-term renal care in DKD. This approach emphasizes modulation of interconnected networks rather than focusing solely on individual molecules, promoting a more integrated perspective on kidney health. Continued integration of mechanistic findings with clinical research will be important to further assess the translational potential of these insights.

## Supplementary Information

Supplementary Material 1.

Supplementary Material 2.

Supplementary Material 3.

Supplementary Material 4.

## Data Availability

No datasets were generated or analysed during the current study.

## Abbreviations

AMPK: Adenosine monophosphate-activated protein kinase
Bax: Bcl-2-associated X protein
CKD: Chronic kidney disease
CMKLR1: Chemokine-like receptor 1
CRH: Corticotropin-releasing hormone
ECM: Extracellular matrix
eGFR: Estimated glomerular filtration rate
ER: Endoplasmic reticulum
eNAMPT: Extracellular nicotinamide phosphoribosyltransferase
FIZZ3: Found in inflammatory zone 3 (Resistin)
GEC: Glomerular endothelial cell
GLP-1RA: Glucagon-like peptide-1 receptor agonist
HMGB1: High mobility group box 1
ICAM-1: Intercellular adhesion molecule-1
IL: Interleukin
IR: Insulin resistance
IRS-1: Insulin receptor substrate-1
JAK/STAT: Janus kinase / signal transducer and activator of transcription
LCN2: Lipocalin-2
LC3: Microtubule-associated protein 1A/1B-light chain 3
MAPK: Mitogen-activated protein kinase
MCP-1: Monocyte chemoattractant protein-1
mTOR: Mechanistic target of rapamycin
NAD⁺: Nicotinamide adenine dinucleotide
NLRP3: NOD-, LRR- and pyrin domain-containing protein 3
NO: Nitric oxide
NGAL: Neutrophil gelatinase-associated lipocalin
NF-κB: Nuclear factor kappa-light-chain-enhancer of activated B cells
PGC-1α: Peroxisome proliferator-activated receptor gamma coactivator 1-alpha
PI3K: Phosphoinositide 3-kinase
PTECs: Proximal tubular epithelial cells
RAS: Renin-angiotensin system
ROS: Reactive oxygen species
SIRT1: Sirtuin 1
Smad: Mothers against decapentaplegic homolog
SGLT2i: Sodium-glucose cotransporter 2 inhibitor
Th: T helper cell
TNF-α: Tumor necrosis factor-alpha
TGF-β: Transforming growth factor-beta
TLR4: Toll-like receptor 4
VEGF: Vascular endothelial growth factor
