# Supplementary material for: Adipokine networks in diabetic kidney disease: mechanistic insights and therapeutic implications
Source: Lipids Health Dis. 2026 Jan 10;25:43. doi: 10.1186/s12944-025-02851-9 (PMC12879388; doi:10.1186/s12944-025-02851-9)
Supplement: Supplementary file 1 — Supplementary Material 1. [file 12944_2025_2851_MOESM1_ESM.pdf]

YIMORE

EDITING CERTIFICATE

To whom it may concern:

This document is to confirm that *Zibo Yimore Translation CO. LTD* happily provided English proofreading services for the following thesis.

Topic: Adipokine Networks in Diabetic Kidney Disease: Mechanistic Insights and Therapeutic Implications

Author: Ke Yang, Yuyang Fang, Junbo He, Jing Li

We performed comprehensive language editing, including corrections in grammar, punctuation, and syntax to the best of our abilities. The revised document was returned to the author on 07/11/2025. We are not aware of any changes made to the manuscript after this date and have kept a copy of the original file for any potential legal dispute.

Sincerely,

Henry Graff

Zibo Yimore Translation CO. LTD

Tel: 0533-2775538 Email:yimorezhls@163.com
