# Supplementary material for: Adipokine networks in diabetic kidney disease: mechanistic insights and therapeutic implications
Source: Lipids Health Dis. 2026 Jan 10;25:43. doi: 10.1186/s12944-025-02851-9 (PMC12879388; doi:10.1186/s12944-025-02851-9)
Supplement: Supplementary file 2 — Supplementary Material 2. [file 12944_2025_2851_MOESM2_ESM.pdf]

iThenticate

972568568971395072.docx

by Shiju Wen

Submission date: 06-Nov-2025 09:34PM (UTC-0600)
Submission ID: 2806191668
File name: 972568568971395072.docx (84.08K)
Word count: 11811
Character count: 81345

iThenticate

iThenticate

Adipokine Networks in Diabetic Kidney Disease: Mechanistic Insights and Therapeutic Implications

Formatted: Shadow

Abstract：Diabetic kidney disease (DKD), the predominant microvascular complication of diabetes mellitus, perpetuates a significant global health and socioeconomic challenge, complicating the pursuit of sustainable renal care. Adipokines, bioactive proteins that modulate lipid metabolism and are secreted by adipose tissue, act as pivotal mediators that integrate systemic metabolic and inflammatory signals with renal pathophysiology. Mechanistic investigations reveal that adipokines orchestrate a range of interconnected pathways, which include metabolic dysregulation (characterized by insulin resistance and lipid overload), immune-inflammatory responses (mediated by nuclear factor kappa B [NF-κB], NLR family pyrin domain containing 3 [NLRP3], and chemokine axes), oxidative stress coupled with mitochondrial dysfunction (involving adenosine monophosphate-activated protein kinase [AMPK] and peroxisome proliferator-activated receptor gamma coactivator 1-alpha [PGC-1α], reactive oxygen species [ROS]), endothelial dysfunction, fibrogenesis (driven by transforming growth factor beta [TGF-β]/Smad and epithelial-mesenchymal transition [EMT]), and the imbalance between apoptosis and autophagy. Protective adipokines such as adiponectin, irisin, and vaspin mitigate harmful signaling, whereas leptin, resistin, visfatin, and chemerin amplify injury through pro-inflammatory, pro-fibrotic, and lipotoxic pathways. Both circulating and urinary levels of adipokines correlate with proteinuria, th

Formatted: Shadow

iThenticate

iThenticate

e decline in estimated glomerular filtration rate, and overall prognosis, underscoring their utility in early detection, risk stratification, and therapeutic monitoring. Recent studies suggest that pharmacological, genetic, and lifestyle interventions may beneficially adjust adipokine networks, thereby offering renal protection. The integration of multi-omics approaches, single-cell analysis, and spatial profiling with models that closely mimic human physiology is essential for identifying key signaling nodes, validating biomarkers, and developing precision-targeted therapies. Collectively, a detailed, network-oriented understanding of lipid-regulating adipokines lays the groundwork for personalized prevention and treatment strategies in DKD, enhancing patient-centered and sustainable approaches to kidney health.

Keywords: Diabetic Nephropathies; Adipokines; Lipid Metabolism; Inflammation; Oxidative Stress; Fibrosis; Systems Biology; Precision Medicine

1. Introduction

Diabetes mellitus (DM) constitutes a globally prevalent chronic metabolic disorder, which is categorized into type 1 diabetes mellitus (T1DM) and type 2 diabetes mellitus (T2DM). Among its complications, diabetic kidney disease (DKD) emerges as one of the most common and severe, afflicting approximately 50% of individuals with T2DM and 33% of those with T1DM [1]. DKD has become the foremost cause of end-stage renal disease (ESRD) and significantly amplifies cardiovascular risk, thereby imposing a substantial clinical and soci

Formatted: Shadow

iThenticate

iThenticate

oeconomic burden. The Global Burden of Disease (GBD) study reports that the age-standardized incidence rate (ASIR) of DKD associated with T2DM increased by 21.0% between 1990 and 2021, whereas the ASIR for DKD due to T1DM saw a rise of 19.3% during the same timeframe [2,3]. These figures are derived from complex modeling of diverse data sources, distinguishing between types of diabetes and adjusted for age structure, yet they continue to face limitations inherent in diagnostic coding and data availability across various regions. Such trends underscore the pressing need for innovative preventive and therapeutic strategies.

The escalating burden of DKD challenges the glucocentric perspective of its pathogenesis and unveils a complex interplay of metabolic, inflammatory, and fibrotic pathways [4]. Adipose tissue (AT), traditionally regarded merely as an energy storage depot, is now recognized as a dynamic endocrine organ. This paradigm shift began with the discovery of leptin in the 1990s [5]. As a crucial metabolic hub, AT secretes an array of bioactive proteins known as adipokines, which include adiponectin, leptin, resistin, and apelin. These molecules exert systemic effects on energy metabolism, insulin sensitivity, immune regulation, vascular homeostasis, redox balance, and fibrotic remodeling [6]. It is notable that adipokine expression and secretion can demonstrate sexual dimorphism and vary according to adipose depot, factors that may contribute to heterogeneity in DKD susceptibility and clinical presentation [7-9].

The current, often fragmented approach to studying individual adipokines d

Formatted: Shadow

iThenticate

iThenticate

oes not sufficiently capture the complexity of their interactions in DKD. Therefore, a holistic, network-oriented perspective is critically needed to bridge the gap between isolated mechanistic insights and the multifactorial nature of the disease. This review aims to provide such a synthesis. While we comprehensively explore the expanded roles of adipokines across metabolic, inflammatory, and fibrotic axes, our primary goal is to promote a more integrated understanding that can facilitate the translation of adipokine biology into novel diagnostic and therapeutic strategies for DKD. Adopting this network-based framework, rather than focusing narrowly on a single pathway, is essential for decoding the systemic nature and heterogeneity of DKD, and represents the innovative aspect of this work.

2. Classification and Network of Adipokines

AT serves as a metabolically active endocrine organ that secretes adipokines, which are crucial in regulating energy metabolism, insulin sensitivity, inflammation, and fibrosis via endocrine, autocrine, and paracrine mechanisms. Adipokines are broadly categorized into classical and emerging groups based on the timeline of their discovery and the extent of their characterization (Table 1).

To elucidate their synergistic effects in DKD, adipokines may be considered interconnected elements within a metabolic-inflammatory signaling network that impacts long-term renal outcomes.

Table 1. Overview of classical and emerging adipokines

Formatted: Shadow

iThenticate

iThenticate

Category	Adipokine	Source/Expression	Core Biological Features/Functions
Classical	Adiponectin	Adipocytes (subcutaneous WAT predominant)	Enhances insulin sensitivity; anti-inflammatory; anti-atherosclerotic
Classical	Leptin	Adipocytes; expression correlates with fat mass	Regulates appetite and energy expenditure; modulates mitochondrial activity; influences inflammation
Classical	Resistin	Macrophages (predominant in humans)	Links obesity to insulin resistance; pro-inflammatory cytokine induction
Classical	Apelin	Adipocytes, multiple organs	Activates AMPK; improves glucose/lipid metabolism; inhibits fibrosis
Emerging	Visfatin (NAMPT/PBEF)	Visceral AT, macrophages	NAD^{+} synthesis (intracellular); pro-inflammatory mediator (extracellular)
Emerging	Vaspin	Visceral AT	Anti-inflammatory; regulates ER stress; inhibits apoptosis
Emerging	Chemerin	Adipocytes, liver	Modulates immunity and metabolism; promotes ECM deposition
Emerging	Irisin	Skeletal muscle, AT	Induces browning of white fat; antioxidant; metabolic improvement
Emergin	Lipocalin-2	Multiple tissues	Inflammation, iron metabolism; early

Formatted: Shadow

iThenticate

iThenticate

Category	Adipokine	Source/Expression	Core Biological Features/Functions
g	(LCN2/NGAL)	(adipocytes, kidney)	biomarker of tubular injury

Note: WAT, white adipose tissue; AMPK, AMP-activated protein kinase; ECM, extracellular matrix; ER stress, endoplasmic reticulum stress; NAD^+, nicotinamide adenine dinucleotide. This table summarizes the classification of classical and emerging adipokines, as well as their primary sources and core biological functions.

2.1 Classical Adipokines

Classical adipokines, such as adiponectin, leptin, resistin, and apelin, are well recognized for their roles in metabolic regulation, inflammation, and the pathogenesis of DKD.

2.1.1 Adiponectin

Adiponectin is predominantly secreted by adipocytes and is characterized by its collagen-like domains and globular regions [10]. It exerts its effects through several receptors, including adiponectin receptor 1/2 (AdipoR1/R2), calreticulin, and T-cadherin [11]. The interaction between its globular domain and T-cadherin not only inhibits atherosclerosis [12] but also promotes exosome biogenesis and reduces ceramide synthesis [13], enhancing insulin sensitivity and mitigating insulin resistance [14, 15]. Collectively, these mechanisms contribute to me

Formatted: Shadow

iThenticate

iThenticate

tabolic and cardiovascular protection.

2.1.2 Leptin

Leptin, synthesized by adipocytes, is modulated by lipid accumulation, LEP gene expression, and cell size. In the hypothalamus, it diminishes the activity of neuropeptide Y (NPY) and gamma-aminobutyric acid (GABA) neurons, elevates corticotropin-releasing hormone (CRH) expression, curtails appetite, and augments energy expenditure [16]. Within adipocytes, leptin boosts mitochondrial activity and fatty acid oxidation [17], while concurrently inhibiting pancreatic insulin secretion [18]. In cases of obesity, leptin resistance compromises these functions, leading to hyperinsulinemia and weight gain. Furthermore, leptin activates nuclear factor kappa B (NF-κB), which promotes inflammation [5] and influences glomerular filtration, potentially facilitating early proteinuria in DKD.

2.1.3 Resistin

Initially identified as a mediator between obesity and insulin resistance, resistin, also known as found in inflammatory zone 3 (FIZZ3), is predominantly secreted by macrophages in humans. It activates NF-κB through Toll-like receptor 4 (TLR4), catalyzing the secretion of pro-inflammatory cytokines such as C-reactive protein (CRP), tumor necrosis factor-alpha (TNF-α), and interleukin-6 (IL-6) [19]. Transgenic mice overexpressing human resistin exhibit exacerbated AT inflammation, increased skeletal muscle lipid accumulation, and compromised insulin signaling under high-fat diet conditions [20].

Formatted: Shadow

iThenticate

iThenticate

2.1.4 Apelin

Apelin, an endogenous ligand for the APJ G protein-coupled receptor, is secreted by adipocytes and various other tissues. The apelin-APJ axis activates adenosine monophosphate-activated protein kinase (AMPK), inhibits the transforming growth factor-beta (TGF-β)/Smad signaling pathway to ameliorate renal interstitial fibrosis [21], and enhances glucose uptake and lipid metabolism through the AMPK, phosphoinositide 3-kinase/protein kinase B (PI3K/Akt), and endothelial nitric oxide synthase (eNOS) pathways [22]. A randomized double-blind trial has demonstrated that exogenous administration of apelin significantly enhances insulin sensitivity in overweight men [23].

Summary

Classical adipokines serve as pivotal regulators in the pathogenesis of DKD, orchestrating metabolic and inflammatory signals between AT and other organs. Their functions provide a foundational mechanism that allows newer adipokines to further diversify and enhance adipose-renal communication within the context of metabolic diseases.

2.2 Emerging Adipokines

Emerging adipokines augment the functions of classical adipokines by modulating metabolism, inflammation, and fibrosis, each assuming distinct and context-dependent roles in DKD.

Formatted: Shadow

iThenticate

iThenticate

2.2.1 Visfatin (nicotinamide phosphoribosyltransferase, NAMPT; pre-B cell colony-enhancing factor, PBEF)

Visfatin operates intracellularly as an NAD^+ synthase (iNAMPT) and extracellularly as a pro-inflammatory cytokine (eNAMPT). The latter activates NF-κB via TLR4, inducing expression of IL-6 and TNF-α, and promoting endothelial dysfunction [24-29]. In DKD, elevated visfatin levels are associated with mesangial cell proliferation, markers of tubular fibrosis, severity of proteinuria, and reduced estimated glomerular filtration rate (eGFR) [30,31].

2.2.2 Vaspin

Vaspin, a serine protease inhibitor, attenuates NF-κB signaling and diminishes the release of pro-inflammatory cytokines [32-34]. It also influences endoplasmic reticulum (ER) stress and lysosomal function, inhibits activation of the NLRP3 inflammasome, and reduces apoptosis in tubular epithelial cells [35].

2.2.3 Chemerin

Chemerin modulates metabolism and immunity via chemokine-like receptor 1 (CMKLR1) [36,37]. In DKD, it activates pro-inflammatory p38 mitogen-activated protein kinase (MAPK) and NF-κB pathways and promotes extracellular matrix deposition and renal fibrosis through TGF-β1/Smad signaling [38-40].

2.2.4 Irisin

Secreted in response to exercise, irisin fosters the browning of WAT and e

Formatted: Shadow

iThenticate

iThenticate

nhances systemic metabolic function [41]. It activates sirtuin 1 (SIRT1)/nuclear factor erythroid 2-related factor 2 (Nrf2) signaling to mitigate oxidative stress [42], inhibits dynamin-related protein 1 (Drp1)-mediated mitochondrial fission, prevents vascular smooth muscle calcification, and has shown renoprotective effects in chronic kidney disease (CKD) models [43].

2.2.5 Lipocalin-2 (LCN2/neutrophil gelatinase-associated lipocalin, NGAL)

Lipocalin-2 is expressed in various tissues and regulates inflammation, iron metabolism, and energy homeostasis. It is an early biomarker of tubular injury [44,45]. In DKD, elevated levels correlate with proteinuria and renal dysfunction [46], and it may have dual effects on fibrosis [47].

Summary

Both classical and emerging adipokines are integral to the metabolic, inflammatory, and fibrotic pathways. Their dysregulated expression in DKD underscores a complex endocrine-mediated network that links AT with kidney function. The subsequent section will delve into their mechanistic roles in the pathogenesis of DKD.

3. Mechanistic Roles of Adipokines in Diabetic Kidney Disease

Accumulating evidence suggests that adipokines play multifaceted roles that extend beyond energy regulation, directly influencing the pathogenesis of DK

Formatted: Shadow

iThenticate

iThenticate

D. These adipokines do not act in isolation but function as interconnected components within a larger signaling network. This network compromises insulin signaling, activates inflammatory and inflammasome pathways, induces oxidative stress, disrupts the balance between apoptosis and autophagy, and promotes fibrosis and tissue remodeling. The subsequent sections elaborate on these mechanisms, emphasizing the crosstalk and integration that characterize the adipokine network as a whole, as well as its tissue-specific impacts on glomeruli, proximal tubules, and the renal microvasculature (refer to Table 2 and Figure 1).

Table 2. Mechanistic roles of adipokines in DKD

(Refer to the end of the document, before the References section)

Figure 1. Mechanistic overview of adipokine-mediated pathways in DKD.

The intricate interplay among adipokines is conceptually summarized through three core, antagonistic signaling axes that integrate their collective effects on renal pathology:

The Energy & Oxidative Stress Axis (AMPK/PGC-1α), modulated by both protective (e.g., adiponectin) and deleterious (e.g., leptin) adipokines.

The Inflammation & Immunity Axis (NF-κB/NLRP3), activated by pro-inflammatory adipokines and suppressed by protective ones.

The Tissue Fibrosis Axis (TGF-β/Smad), driven by pro-fibrotic adipokines and inhibited by anti-fibrotic agents.

The dynamic equilibrium between these opposing networks at shared signaling nodes ultimately dictates the renal outcomes in DKD. This figure was gene

Formatted: Shadow

iThenticate

iThenticate

rated using BioGDP.com [48] (license number: GDP2025O2HEB2).

3.1 Metabolic Dysregulation

3.1.1 Impaired Insulin Signaling

Impaired insulin signaling constitutes an early and crucial mechanism in the pathogenesis of DKD. Multiple adipokines are implicated in this impairment. Resistin, for instance, induces ER stress and activates c-Jun N-terminal kinase (JNK) and NF-κB, leading to phosphorylation of IRS-1 at Ser307, which inhibits Akt and eNOS activity, thereby exacerbating insulin resistance in renal cells [49]. Chemerin activates p38 MAPK and NF-κB, reduces phosphorylation of IRS-1/Akt, impairs glucose uptake, and is associated with clinical manifestations of insulin resistance [50,51]. Elevated leptin levels, often found in obesity and hyperinsulinemia, upregulate TGF-β1 and vascular endothelial growth factor (VEGF), which promote glomerular hypertrophy and fibrosis [52]. Conversely, adiponectin enhances insulin signaling through AMPK and PI3K/Akt pathways, mitigating oxidative stress and inflammation; its deficiency is associated with podocyte injury and proteinuria [53]. The ratio of leptin to adiponectin has been proposed as a marker of the metabolic-inflammatory status in DKD [54].

3.1.2 Lipid Overload

Excessive lipid accumulation directly contributes to renal injury, extending beyond its impact on insulin resistance. Visfatin and chemerin upregulate the s

Formatted: Shadow

iThenticate

cavenger receptor CD36, which promotes lipid deposition, activates NF-κB, and increases expression of MCP-1 and TGF-β1. These changes lead to macrophage infiltration and fibrogenesis, often accompanied by mitochondrial dysfunction [55]. Clinically, elevated serum levels of visfatin are correlated with proteinuria and reduced renal function [56].

3.1.3 Energy Sensing Dysregulation

Dysregulation in energy sensing exacerbates metabolic injury significantly. The signaling pathway of adiponectin-AMPK enhances fatty acid β-oxidation, inhibits the activation of mTOR, reduces the production of reactive oxygen species (ROS), and preserves nephron integrity [57,58]. In contrast, elevated levels of visfatin suppress SIRT1 activity, impair mitophagy, and disrupt energy homeostasis, thereby aggravating renal damage [59].

3.1.4 Integrated Adipokine Network and Net Effect

Adipokines function within a dynamic network that exhibits both opposing and synergistic effects. Adiponectin attenuates the detrimental impacts of resistin and leptin, while chemerin and visfatin intensify lipotoxic and glucotoxic stress. This interaction contributes to the clinical heterogeneity observed in DKD and may affect therapeutic responses, indicating that strategies should focus on restoring the balance of the entire network rather than targeting isolated factors.

Formatted: Shadow

iThenticate

3.2 Inflammation and Immune Dysregulation

3.2.1 Central Role of Inflammation

A chronic, low-grade inflammatory state is a primary driver of DKD, with adipokines coordinating both local and systemic immune responses that exacerbate disease progression.

3.2.2 Immune Cell Recruitment and Polarization

Chemerin, which is upregulated in the kidneys during DKD, promotes chemotaxis of monocytes and dendritic cells through ChemR23 [60]. Leptin facilitates the differentiation of $CD4^{+}$ T-cells into pro-inflammatory Th1 and Th17 subtypes and promotes M1 macrophage polarization, thereby enhancing the production of IL-6 and TNF-α and linking metabolic disturbances to immune activation [61]. Resistin provokes peripheral monocytes to secrete IL-1β and TNF-α, and it enhances endothelial adhesion, thereby facilitating leukocyte recruitment to renal tissues [62].

3.2.3 Inflammasome and Signaling Pathway Activation

Chemerin exacerbates tubular pro-inflammatory responses by activating the MAPK and NF-κB pathways, leading to increased cytokine production and localized inflammation [63]. Lipocalin-2 triggers the NLRP3 inflammasome via the HMGB1-TLR4 axis, inducing the maturation and release of IL-1β and IL-18. This mechanism contributes to renal fibrosis and underscores the crosstalk betw

Formatted: Shadow

iThenticate

iThenticate

een inflammation and tissue remodeling mediated by adipokines [64,65].

3.2.4 Protective Adipokines

Adiponectin inhibits NF-κB activation via the AMPK and PPARα pathways, thereby reducing the production of TNF-α and IL-1β [52]. Vaspin mitigates ERK/NF-κB signaling in tubular cells, diminishing the expression of pro-inflammatory factors and counterbalancing the effects of pro-inflammatory adipokines [66].

3.2.5 Network Regulation and Metabolic Interplay

Adipokines operate within a complex network, featuring feedback loops that integrate metabolic and inflammatory signals. For example, leptin induces TNF-α in a positive feedback loop, which is mitigated by adiponectin's effects. Concurrently, the NF-κB-mediated inhibition of IRS-1/PI3K/Akt signaling exacerbates insulin resistance, creating a deleterious cycle central to the progression of DKD and influenced by the overall state of the adipokine network [67,68].

3.3 Oxidative Stress and Mitochondrial Dysfunction

3.3.1 Excessive ROS and Impaired Antioxidant Defense

Oxidative stress constitutes a fundamental pathogenic mechanism in DKD, significantly influenced by adipokines that contribute to renal injury. Under conditions of hyperglycemia, disruptions in glucose and lipid metabolism coincide with protein kinase C (PKC) activation, leading to a substantial increase in the

Formatted: Shadow

iThenticate

iThenticate

production of ROS within renal cells [69,70]. This increased mitochondrial workload in mesangial cells, podocytes, and tubular epithelial cells, accompanied by a simultaneous decline in antioxidant defenses, results in sustained oxidative damage.

3.3.2 Mitochondrial Structural and Functional Impairment

Persistent oxidative stress inflicts direct damage on mitochondria, compromising their structural integrity and bioenergetic functionality. This damage is characterized by a reduced membrane potential, impaired ATP synthesis, mutations in mitochondrial DNA, and dysregulated autophagy [71,72]. Importantly, these compromised mitochondria release mitochondrial damage-associated molecular patterns (mtDAMPs), which activate innate immune receptors such as TLRs and the NLRP3 inflammasome, thereby initiating a feed-forward loop that exacerbates inflammation and fibrosis [73].

3.3.3 Adipokine-Mediated Regulation of Oxidative Stress

Adipokines play critical and contrasting roles in regulating oxidative balance. Protective adiponectin activates the AMPK/PGC-1α pathway, enhancing mitochondrial biogenesis, inhibiting NADPH oxidase activity, and thus maintaining mitochondrial health [53,74]. Conversely, leptin promotes ROS generation through NADPH oxidase and stimulates the PI3K/Akt and JAK/STAT pathways, intensifying oxidative damage [75-77]. Similarly, chemerin augments ROS production and inflammatory responses via its CMKLR1 receptor and downstream p38 M

Formatted: Shadow

iThenticate

iThenticate

APK signaling [38,78], while lipocalin-2 further disrupts mitochondrial equilibrium [79]. In contrast, irisin mitigates oxidative stress by activating the AMPK/SIRT1/PGC-1α pathway, thereby enhancing mitochondrial function and reducing harmful ROS accumulation [80,81].

3.3.4 Metabolic-Inflammatory Cross-talk via Oxidative Stress

Oxidative stress and inflammation interact synergistically, reinforcing each other and accelerating the progression of DKD. Pro-inflammatory adipokines, such as chemerin and resistin, increase NADPH oxidase activity, raising ROS levels which, in turn, activate the NF-κB pathway. This activation establishes a positive feedback loop that perpetuates inflammation. Concurrently, leptin not only enhances ROS production but also reduces cellular antioxidant capacity, thereby impeding ROS clearance and further exacerbating renal damage [76,82].

3.4 Endothelial Dysfunction and Microvascular Damage

3.4.1 Pathophysiological Basis

Endothelial dysfunction is a principal factor driving the progression of DKD. Glomerular endothelial cells (GECs) are critical in maintaining vascular tone and the integrity of the filtration barrier. In diabetes, the combined effects of hyperglycemia, oxidative stress, and chronic inflammation collectively diminish nitric oxide (NO) availability, increase vascular permeability, and induce endothelial damage, which ultimately leads to microvascular lesions [83].

Formatted: Shadow

iThenticate

iThenticate

3.4.2 Endothelial Activation and Structural Remodeling

Endothelial dysfunction is manifested by impaired eNOS activity under conditions of hyperglycemia and insulin resistance, which decreases NO synthesis. Elevated levels of adhesion molecules, such as ICAM-1 and VCAM-1, facilitate the adhesion and transmigration of leukocytes. Persistent endothelial activation leads to apoptosis and structural alterations, including thickening of the basement membrane [84,85].

3.4.3 Adipokine-Mediated Regulation of Endothelial Function

Adipokines exhibit dichotomous effects on endothelial homeostasis.

Protective adipokines, such as adiponectin and irisin, enhance NO availability through AMPK-dependent eNOS activation and inhibit NF-κB signaling, thereby preserving vascular function [86-88].

Conversely, pro-inflammatory adipokines contribute to endothelial dysfunction: leptin upregulates adhesion molecules through the JAK2/STAT3 and PI3K/Akt pathways; chemerin enhances ROS production and inflammatory signaling through ERK1/2 and p38 MAPK; and lipocalin-2 impairs mitochondrial function and increases vascular permeability [36,89-92].

3.4.4 Consequences of Endothelial Injury

Persistent endothelial damage accelerates the formation of microvascular lesions, leading to renal ischemia and hypoxia. This, in turn, activates the hypoxia-inducible factor 1-alpha (HIF-1α) and TGF-β pathways. While pro-inflammato

Formatted: Shadow

iThenticate

iThenticate

ry adipokines exacerbate this cycle of injury, protective adipokines counteract these effects and maintain vascular integrity, thus critically influencing the progression of DKD [93].

3.5 Glomerular-Tubular Injury and Renal Interstitial Fibrosis

3.5.1 Pathological Progression

In DKD, injury to the glomeruli and proximal tubules is synergistic and mutually reinforcing, which accelerates renal interstitial fibrosis and functional decline. Hyperglycemia induces mesangial matrix expansion, capillary loop disorganization, and disruption of the podocyte slit diaphragm, collectively compromising the filtration barrier [94]. Concurrently, proximal tubular epithelial cells (PTECs), highly susceptible to glucotoxicity and proteinuric stress, exhibit mitochondrial dysfunction, lysosomal stress, and ferroptosis, further exacerbating renal injury [95,96].

3.5.2 Fibrogenic Mechanisms

Renal interstitial fibrosis is propelled by multiple convergent pathways, including the epithelial-to-mesenchymal transition (EMT) of PTECs, mesangial cell activation, and excessive extracellular matrix (ECM) deposition. Transforming growth factor-beta1 (TGF-β1) plays a pivotal role, activating Smad2/3 signaling to promote the expression of pro-fibrotic genes. Hypoxia further enhances TGF-β signaling through upregulation of HIF-1α, establishing a persistent positive

Formatted: Shadow

iThenticate

iThenticate

feedback loop that perpetuates fibrosis [97].

3.5.3 Adipokine-Mediated Regulation of Fibrosis

Adipokines significantly influence fibrogenic pathways, displaying both pathogenic and protective roles.

Pro-fibrotic adipokines include leptin, which upregulates the TGF-β receptor II (TβRII) in mesangial cells through PI3K signaling, promoting collagen and fibronectin deposition [98]; resistin, which activates TLR4/p65 signaling, enhancing angiotensinogen production and thereby augmenting RAS activation and inflammation [99,100]; chemerin, which intensifies TGF-β1/Smad/CTGF signaling, while receptor antagonism reduces collagen accumulation [39]; and lipocalin-2, which exacerbates mitochondrial injury, ROS production, and apoptosis, worsening fibrosis [79].

Anti-fibrotic adipokines include adiponectin, which inhibits TGF-β, NF-κB, and MCP-1 pathways, delaying fibrosis and preserving renal architecture [101]; and irisin, which suppresses TGF-β1/Smad4 and β-catenin signaling, reverses EMT, and mitigates interstitial fibrosis [102].

3.5.4 Integrated Effects and Therapeutic Implications

Collectively, adipokines form a complex regulatory network that modulates glomerular-tubular injury and interstitial fibrosis through overlapping and opposing signaling axes. Their dual roles, pathogenic and protective, therefore provide a mechanistic framework for understanding the heterogeneity of DKD and h

Formatted: Shadow

iThenticate

iThenticate

ighlight promising pathways for novel therapeutic strategies.

3.6 Imbalance Between Apoptosis and Autophagy

3.6.1 Pathophysiological Basis

In DKD, renal cells endure persistent metabolic insults due to hyperglycemia, oxidative stress, and chronic inflammation. This hostile microenvironment disrupts cellular homeostasis, resulting in a pathological imbalance between cell death and survival mechanisms. A significant increase in apoptotic cell death occurs simultaneously with the suppression of the protective autophagy pathway, collectively exacerbating structural damage and the loss of functional nephrons [103,104].

3.6.2 Key Dysregulated Pathways

Apoptosis Activation: In DKD, the intrinsic (mitochondrial) apoptotic pathway predominantly mediates cell death. Mitochondrial outer membrane permeabilization is induced by ROS and glucose toxicity, characterized by the upregulation of Bax and the downregulation of Bcl-2, which leads to 32 the activation of the caspase cascade and programmed cell death. Furthermore, ER stress, triggered by glucotoxicity, intensifies tubular apoptosis [103,104].

Autophagy Suppression: Under diabetic conditions, the essential cellular clearance mechanism of autophagy is compromised. This impairment is evidenced by reduced levels of key autophagy markers such as Beclin-1 and LC3-II, alo

Formatted: Shadow

iThenticate

iThenticate

ngside increased activity of the mechanistic target of rapamycin (mTOR). The accumulation of damaged mitochondria, protein aggregates, and other deleterious cellular components not only impairs cell function but also intensifies apoptotic signaling, perpetuating a cycle of injury [105].

3.6.3 Adipokine-Mediated Regulation of Cell Fate

Adipokines are critical extracellular signals that regulate the balance between apoptosis and autophagy within the diabetic kidney.

Protective Adipokines: Adiponectin activates AMPK signaling, which subsequently inhibits mTOR activity. This dual action enhances autophagic flux and diminishes ROS-induced apoptosis, thereby offering protection to renal cells [53].

Deleterious Adipokines: Leptin promotes apoptosis in podocytes and contributes to mesangial cell hypertrophy through the activation of JAK/STAT and PI3K/Akt signaling pathways [106]. Lipocalin-2 increases mTOR activity and induces DRP1-dependent mitochondrial fragmentation, effects that can be counteracted by the pharmacological inhibition of mTOR with rapamycin [79].

3.6.4 Integrated Effects and Pathological Implications

The interactions among adipokines form a regulatory network that decisively influences the fate of renal cells. The predominance of pro-apoptotic signals (e.g., from leptin and lipocalin-2) over pro-survival and pro-autophagy signals (e.g., from adiponectin) drives nephron injury in DKD. This disruption of cellu

Formatted: Shadow

iThenticate

iThenticate

lar homeostasis not only leads directly to cell loss but also contributes to the progression of renal dysfunction. Consequently, therapeutic strategies aimed at restoring this balance, either by inhibiting apoptosis or inducing protective autophagy, offer a promising approach to preserving renal structure and slowing disease progression.

4. Controversies and Divergences

Adipokines are recognized as pivotal regulators in DKD; however, significant inconsistencies concerning their expression, mechanisms, and clinical relevance impede their utilization as biomarkers or therapeutic agents. The primary sources of these divergences include interspecies variations, stages of disease progression, cellular environments, and comorbid conditions in patients.

4.1 Heterogeneity of Expression and Clinical Associations

The clinical implications of many adipokines are known to be context-sensitive. Visfatin, for instance, has been identified as elevated and associated with proteinuria in certain patient cohorts [56], yet it exhibits weak or no correlation with the decline in eGFR in other groups, where it is instead linked to systemic inflammation [107]. Preclinical studies occasionally propose protective roles for visfatin [108], adding complexity to its interpretation. Similar ambiguities are noted with chemerin and vaspin. Extrinsic metabolic factors, such as acute hyperbilirubinemia, which is implicated in early tubular injury even in mild c

Formatted: Shadow

iThenticate

iThenticate

onditions, may independently influence renal damage or adipokine signaling. This underscores the intricate pathophysiological context within which adipokines function [109], necessitating meticulous control for these variables to ensure accurate clinical interpretation.

4.2 Mechanistic Inconsistencies, Tissue Specificity, and Translational Insights

Adipokines demonstrate tissue- and context-dependent effects. For example, adiponectin predominantly exerts anti-inflammatory and antifibrotic actions in tubular cells through AMPK/PPARα-mediated inhibition of TGF-β [110]. However, under lipopolysaccharide (LPS) stimulation, adiponectin can induce pro-inflammatory responses in HK-2 cells [111]. Chemerin facilitates the recruitment of dendritic and T cells [112], while vaspin reduces inflammation via modulation of ER stress [66]. Variations in cell type, temporal dynamics, and experimental conditions contribute to significant discrepancies in results.

Emerging technologies, such as single-cell and spatial transcriptomics, enable the mapping of adipokine receptor expression across diverse renal cell types, including podocytes, mesangial cells, and tubular cells. Integrating these spatially resolved datasets with multi-omics approaches (including transcriptomics, proteomics, and metabolomics) throughout different stages of disease facilitates the identification of crucial regulatory nodes that dictate biological outcomes. Such insights are instrumental in refining patient stratification and developing bioma

Formatted: Shadow

iThenticate

iThenticate

rker-driven therapeutic strategies, such as distinguishing between tubular injury-dominant and inflammation-predominant DKD for targeted adipokine signaling modulation.

4.3 Limitations of preclinical models

The majority of mechanistic insights are derived from rodent models of obesity and diabetes, such as db/db and ob/ob mice, which exhibit metabolic and renal pathologies distinct from human DKD. Species-specific variations in adipokine expression, processing, and splicing, exemplified by irisin, pose additional challenges to translational efforts [113,114]. Human-relevant experimental platforms, including kidney organoids, precision-cut tissue slices, and ex vivo human samples, are crucial for the validation of mechanisms and therapeutic targets.

4.4 Neglected network complexity

Most research studies assess adipokines in isolation, neglecting the complex regulatory networks that govern their collective effects. In reality, adipokines often function synergistically or antagonistically through shared signaling pathways such as PI3K/Akt, AMPK, and SIRT1, as well as through the cross-regulation of inflammatory and fibrotic mediators [115-117]. For example, leptin promotes the secretion of TNF-α and IL-6, which in turn influence the expression of chemerin, while adiponectin and visfatin modulate the PI3K/Akt and ER stress pathways, respectively. Overlooking these interactions can lead to oversimplifi

Formatted: Shadow

iThenticate

iThenticate

ed interpretations of mechanisms and diminish translational relevance. To systematically illustrate these context-dependent effects and address the ongoing debates, we summarize the key controversies and future research priorities for major adipokines in Table 3.

Integrating network-oriented analyses with multi-omics datasets facilitates the identification of central regulatory hubs and aids in the prioritization of combination interventions. Models that are relevant to human physiology, such as kidney organoids or precision-cut tissue slices, can validate these network-level hypotheses under the pathophysiological conditions of DKD.

Table 3. Sources of controversy and proposed resolution pathways for selected adipokines in DKD.

**Adipokine**	**Reported Effect in DKD**	**Species/Model**	**Experimental Context**	**Net Outcome**	**Notes / Future Research**
Adiponectin	Anti-inflammatory, anti-fibrotic	Human tubular cells	Baseline conditions	Protective	Under LPS stimulation: pro-inflammatory; future: integrate human biopsy + organoid studies; stratify patients by receptor expression
Chemerin	Pro-inflammatory, promotes	Rodent DKD models	Variable	Context-depende	Resolve via multi-omics, cell-type

Formatted: Shadow

iThenticate

iThenticate

Adipokine	Reported Effect in DKD	Species/Model	Experimental Context	Net Outcome	Notes / Future Research
	dendritic/T-cell recruitment			nt	-specific and spatial transcriptomics; patient stratification
Vaspin	Anti-inflammatory, ER stress modulation	Rodent DKD models	Variable	Protective	Assess human tubular and mesangial cells; consider receptor profiling; integrate organoid validation
Leptin	Induces TNF-α/IL-6, pro-fibrotic	Rodents & humans	Chronic hyperglycemia	Detrimental	Explore combination therapy with anti-inflammatory modulators; validate network interactions
Visfatin	Variable; sometimes pro-inflammatory, sometimes protective	Human cohorts, db/db mice	Obesity, inflammation	Context-dependent	Stratify by patient phenotype; integrate urinary/serum biomarker profiling; organoid and biopsy validation

Formatted: Shadow

iThenticate

iThenticate

Adipokine	Reported Effect in DKD	Species/Model	Experimental Context	Net Outcome	Notes / Future Research
Irisin	Renoprotective in preliminary studies	Rodent DKD models	Exercise / pharmacological induction	Protective	Validate in human organoids and biopsy tissue

Notes:

Abbreviations: DKD, diabetic kidney disease; LPS, lipopolysaccharide.

Future research priorities focus on leveraging human-relevant models (e.g., organoids, precision-cut tissue slices) and integrating multi-omics to elucidate context-specific effects and enable patient stratification.

4.5 Gaps in clinical application and standardization

Despite the biological complexity of adipokine networks, substantial gaps in clinical application and standardization remain evident. There is a lack of consensus on reference ranges, risk thresholds, or criteria for interventions based on adipokine measurements. Large-scale prospective cohorts utilizing standardized assays are essential to establish the prognostic utility of adipokines. Pharmacological interventions targeting adipokines, such as vaspin and irisin, are in their nascent stages and face challenges related to bioavailability, targeted delivery, and safety, which currently limit their clinical applicability.

4.6 Sex and Body Composition as Sources of Heterogeneity

Moreover, intrinsic biological factors such as sexual dimorphism and patter

Formatted: Shadow

iThenticate

iThenticate

ns of body fat distribution are significant, yet frequently overlooked, sources of heterogeneity in adipokine research. The circulating 36 levels of key adipokines, such as adiponectin and leptin, vary between males and females and are affected by factors like menopausal status and the specific adipose depots, potentially leading to disparate clinical correlations and susceptibility to DKD across sexes [7-8]. Future epidemiological and mechanistic studies need to integrate these variables to refine our understanding of adipokine actions in DKD.

5. Therapeutic Implications

The adipokine network serves as a dynamic interface between metabolism and renal pathology, presenting both a source of biomarkers and a platform for therapeutic intervention in DKD. However, transitioning from mechanistic insights to clinical applications requires a precision medicine approach. This chapter details how employing network-oriented and multi-omics strategies can bridge this gap, facilitating risk stratification, targeted therapies, and ultimately, enhanced renal outcomes.

5.1 Risk Assessment and Early Detection

Circulating and urinary adipokine levels serve as reflections of DKD severity and progression, offering potential biomarkers for early risk stratification. Elevated levels of chemerin and leptin are associated with systemic low-grade inflammation and are predictive of a decline in glomerular filtration rates [118].

Formatted: Shadow

iThenticate

iThenticate

Conversely, urinary adiponectin acts as an early marker of renal injury and responsiveness to treatment [57]. The incorporation of these adipokines into multi-marker panels, possibly integrated with transcriptomic or proteomic profiles, could enhance both the sensitivity and specificity of early detection methods. From a mechanistic standpoint, these biomarkers encapsulate the cumulative effects of metabolic dysregulation, inflammation, and fibrotic signaling, thereby facilitating a comprehensive assessment of DKD risk. Multi-omics approaches and network analyses further support the identification of patient subgroups who might benefit from tailored interventions.

5.2 Therapeutic Targeting of Adipokines

Preclinical studies demonstrate that the direct modulation of adipokine signaling has the potential to mitigate DKD pathology. The administration of recombinant adiponectin or its agonists has been shown to activate AMPK and PI3K/Akt signaling pathways, which in turn reduce tubular oxidative stress, inflammation, and fibrosis [119]. Inhibition of chemerin decreases inflammation in glomerular endothelial cells and reduces extracellular matrix deposition [38]. Preliminary investigations into visfatin inhibitors and irisin analogs indicate renoprotective effects [108,120]. These therapeutic interventions exemplify precision targeting of adipokine networks, providing mechanistically informed strategies for treatment. Key candidates, their mechanisms of action, and approaches for patient stratification aligned with these strategies are synthesized in Table 4.

Formatted: Shadow

iThenticate

iThenticate

(Table 4. Strategies for targeting adipokine networks in DKD.)

Category	Intervention	Targeted Adipokines	Regulated Pathways / Mechanisms	Renal Effects	Precision Patient Stratification
**Established & Repurposed Therapies**	GLP-1R Agonists (GLP-1RA)	↑Adiponectin, ↓Leptin, ↓Resistin	AMPK, PI3K/Akt, anti-inflammatory signaling	Reduce tubular oxidative stress, inflammation, fibrosis	Patients with low adiponectin/high leptin; early or obesity-related DKD [120-121]
	SGLT2 Inhibitors (SGLT2i)	↓Pro-inflammatory adipokines	AMPK, metabolic homeostasis, oxidative stress pathways	Promote renal metabolic homeostasis; reduce inflammation	T2DM patients with DKD; early metabolic dysregulation (multi-omics identified) [123]
**Lifestyle & Adjunctive Strategies**	Lifestyle Interventions (exercise, weight loss, Mediterra	↑Adiponectin, ↓Leptin, ↓Chemerin	AMPK, SIRT1, anti-inflammatory & anti-fibrotic pathways	Improve metabolic-immune homeostasis; reduce systemic inflammat	Early-stage DKD, overweight/obese patients; stratified by baseline adipokine profile [122-124]

Formatted: Shadow

iThenticate

iThenticate

Category	Intervention	Targeted Adipokines	Regulated Pathways / Mechanisms	Renal Effects	Precision Patient Stratification
	nean diet)			ion	
Emerging Biologics & Targeted Therapies	Adiponectin Agonists / Analogs	↑Adiponectin	AMPK, PI3K/Akt	Reduce oxidative stress, inflammation, fibrosis	High-risk DKD patients, multi-omics-guided selection [118]
	Chemerin Inhibition	Chemerin	NF-κB, endothelial activation	Reduces glomerular inflammation, ECM deposition	Patient subgroups with strong inflammatory signatures [38]
	Leptin Blockade	Leptin	TNF-α/IL-6, pro-fibrotic signaling	Attenuates glomerular injury	Patients with confirmed hyperleptinemia; combination therapy [89, 90]
	Irisin Analog Indu	Irisin	AMPK, mitochondrial function	Improves renal metabol	Validate in pilot studies; patients

Formatted: Shadow

iThenticate

iThenticate

Category	Intervention	Targeted Adipokines	Regulated Pathways / Mechanisms	Renal Effects	Precision Patient Stratification
	ction			ism and fibrosis	with mitochondrial dysfunction [80, 81, 102]
	Vaspin Modulation	Vaspin	ER stress, anti-inflammatory	Attenuates tubular/mesangial inflammation	Receptor profiling; combined multi-omics studies [66]
	Visfatin Modulation	Visfatin	NAD+-dependent pathways, inflammatory modulation	Context-dependent (protective vs pro-inflammatory)	Stratify by patient phenotype & biomarker panels [108, 119]
**Next-Generation Platforms**	Nanoparticle-based Delivery / Organoid-guided Therapy	Specific adipokines	Targeted delivery, enhanced bioavailability	Improve renal targeting and therapeutic efficacy	Advanced DKD; poor drug distribution; refined by single-cell/spatial transcriptomics

Notes:

Formatted: Shadow

iThenticate

iThenticate

Integration with multi-omics techniques, encompassing transcriptomics, proteomics, and metabolomics, is crucial for identifying 44 patient subgroups most likely to benefit from these therapeutic interventions. Single-cell and spatial transcriptomics could further elucidate the distribution of adipokine receptors, enhancing the precision of targeting. The potential for combination therapies to yield synergistic effects should be explored. To achieve clinical validation, large-scale prospective studies are indispensable.

5.3 Adjunctive strategies with established treatments

The current management of DKD predominantly employs 15 renin-angiotensin system (RAS) blockers, sodium-glucose cotransporter 2 inhibitors (SGLT2is), and glucagon-like peptide-1 receptor agonists (GLP-1RAs). Although these interventions prove effective, a residual risk remains in certain patient subgroups. GLP-1RAs enhance glycemic control and alter adipokine profiles by increasing circulating adiponectin levels and decreasing leptin and resistin levels, which may attenuate renal inflammation, oxidative stress, and fibrosis [121,122]. Similarly, SGLT2is have demonstrated an ability to raise adiponectin and reduce leptin levels, suggesting their indirect renoprotective effects stem from improved metabolic and inflammatory homeostasis [123]. These established therapies serve as broad network modulators, restoring balance to the dysregulated adipokine signaling landscape rather than targeting a single molecule. This broad modulation supports the rationale for patient stratification based on adipokine network signatu

Formatted: Shadow

iThenticate

iThenticate

res. For example, individuals presenting a 'high-leptin/resistin, low-adiponectin' profile, indicative of a pronounced metabolic-inflammatory drive, may particularly benefit from the adipokine-modulating effects of GLP-1RAs or SGLT2is.

Integration of multi-omics and network-oriented analyses assists in identifying patient subgroups likely to benefit most from these therapies. For instance, individuals characterized by low adiponectin/high leptin levels or elevated chemerin, as identified through transcriptomic or proteomic profiling, may respond preferentially to a combination of GLP-1RA and lifestyle interventions. 57 Lifestyle modifications, such as structured exercise, weight reduction, and adherence to a Mediterranean-style diet, further modulate adipokine networks, enhance metabolic-immune balance, and support early-stage DKD management [124-126]. Coordinated pharmacologic and lifestyle strategies provide a practical approach to translate mechanistic insights into precision medicine applications, facilitating biomarker-guided, individualized therapy.

5.4 Challenges and Future Directions

30

Despite promising preclinical and early clinical results, therapies targeting adipokines face significant challenges, including tissue specificity, rapid degradation or poor bioavailability of peptides, and incomplete characterization of downstream signaling pathways. Targeting stable and druggable nodes such as AMPK, SIRT1, or phosphoinositide 3-kinase/protein kinase B (PI3K/Akt) may alleviate some of these limitations. The use of nanoparticle-mediated delivery, organo

Formatted: Shadow

iThenticate

iThenticate

id, or kidney-on-a-chip platforms could enhance mechanistic validation and tissue-specific targeting, facilitating rational therapeutic design.

Future research should incorporate human kidney biopsy-based multi-omics alongside in vitro and ex vivo models to refine adipokine-targeted approaches. Single-cell and spatial transcriptomics could map the distribution of adipokine receptors across renal cell types such as podocytes, mesangial cells, and tubular epithelial cells, enabling patient stratification and predictive biomarker-guided therapy. Patients could be stratified not only by clinical phenotype but also by molecular signatures, such as a ‘high-leptin/resistin, low-adiponectin’ profile indicative of a strong metabolic-inflammatory drive, to prioritize them for specific adjunctive or targeted therapies. Moreover, integrating biological variables such as sex and body composition into these models is critical, given their established impact on adipokine biology [7-8]. For example, the more favorable adipokine profile observed in premenopausal females may influence disease progression and therapeutic responses, indicating that sex-specific treatment thresholds or strategies may be necessary [7].

5.5 Translation into clinical practice

To advance adipokine-based interventions, extensive multicenter, prospective studies are essential. These studies should encompass a variety of DKD subtypes, stages, and patient demographics, and must be adequately powered to facilitate sex-specific analyses. It is crucial that such studies routinely collect data

Formatted: Shadow

iThenticate

iThenticate

on body composition and fat distribution, in addition to measuring circulating adipokine levels. The incorporation of adipokines into multimarker predictive models holds the potential to enhance risk stratification, improve the early detection of disease, and monitor therapeutic responses more effectively. For instance, integrating clinical data with adipokine profiles and molecular signatures derived from transcriptomics may facilitate the classification of patients into specific subtypes, such as 'tubular-injury-predominant' and 'inflammatory-signature' DKD. This classification could guide the selection of therapies that either target adipokine-mediated inflammation or address metabolic dysregulation. When combined with mechanistic validation from human-relevant platforms and network-level analyses, these strategies could underpin a new paradigm of precision medicine in DKD, moving away from a generic approach to one that is predictive, preventative, and personalized.

Summary

This review has thoroughly explored the significant roles of adipokines as central mediators and modulators in the pathogenesis of DKD. We have detailed the progression from dysregulated metabolic signaling and inflammatory activation to oxidative stress, endothelial dysfunction, and fibrosis, demonstrating how adipokine networks synthesize these fundamental pathological axes. Despite existing controversies regarding the context-specific actions of adipokines, the adoption of network-oriented and multi-omics approaches offers a definitive route

Formatted: Shadow

iThenticate

iThenticate

forward. These methodologies aim to resolve existing conflicts and identify key regulatory hubs. The translation of this mechanistic understanding into clinical practice is now progressing, advancing through risk stratification with biomarker panels and the repurposing of existing metabolic therapies towards pioneering biologics and precision-targeted interventions. The future of DKD management will likely rely on embracing this complexity, transitioning from a uniform treatment strategy to a dynamic, network-informed model of precision medicine that holds promise for preserving renal function and enhancing patient outcomes.

6.Strengths and limitations

6.1 Strengths

This review presents a network-based and integrative perspective on adipokine biology within the context of DKD, effectively bridging molecular mechanisms with translational opportunities. It synthesizes dispersed findings across metabolism, inflammation, oxidative stress, and inter-organ communication into a coherent mechanistic framework, highlighting potential early diagnostic and targeted intervention points.

Another notable strength is its translational and patient-centered focus. The review systematically connects mechanistic insights to current and emerging interventions, including pharmacological agents, biological modulators, and nanotechnology-based strategies, outlining practical approaches toward the precision m

Formatted: Shadow

iThenticate

iThenticate

anagement of DKD. Moreover, by embracing a comprehensive and network-based perspective, this review transcends the typical fragmented narrative of individual adipokines. This approach, although it may limit the depth of discussion on any single molecule, is crucial as it reflects the multifactorial and interconnected nature of DKD pathophysiology, offering a more realistic and clinically relevant framework. The integration of systems biology and multi-omics approaches further augments the potential for individualized risk prediction and therapy optimization.

These attributes highlight this review as a timely and integrative contribution to the dynamic field of DKD research.

6.2 Limitations

Despite its comprehensive scope, this review acknowledges several limitations. Much of the current evidence is associative, derived from small-scale or cross-sectional studies that utilize inconsistent assay methods and feature limited ethnic diversity. Consequently, establishing causal relationships between specific adipokines and renal injury mechanisms necessitates further longitudinal and mechanistic studies.

Additionally, the complexity and context-dependent nature of adipokine signaling challenge the generalization of findings across different disease stages or patient subgroups. Experimental models may not accurately replicate human pathophysiology, highlighting the need for more human-relevant systems, such as

Formatted: Shadow

iThenticate

iThenticate

organoids and spatial multi-omics profiling, to enhance translational accuracy.

Moreover, while this review identifies promising therapeutic targets, clinical translation remains nascent. Future research should focus on the standardization of biomarkers, multicenter validation, and the integration of molecular data into clinical decision-making frameworks to facilitate earlier detection and more effective interventions.

Collectively, these limitations delineate the next steps necessary for achieving biologically grounded, patient-centered, and clinically meaningful advancements in the management of DKD.

7.Conclusion

Adipokines serve as crucial molecular intermediaries between metabolic dysfunction and renal injury in DKD. This review synthesizes evidence from metabolic, inflammatory, and fibrotic pathways, offering a network-based framework that connects basic discoveries with potential clinical applications.

Mechanistic insights into adipokine signaling elucidate how imbalances within this network contribute to glomerular, tubular, and interstitial injury. Understanding these interactions facilitates the early identification of high-risk individuals and enhances opportunities for biomarker-guided diagnosis, risk stratification, and therapeutic monitoring.

These insights are directly clinically relevant, as they translate molecular discoveries into practical decision-making tools for the management of DKD. Cli

Formatted: Shadow

iThenticate

iThenticate

nically, adipokine profiles may augment existing diagnostic panels and aid in tailoring treatment decisions, for example, guiding the selection or combination of SGLT2 inhibitors, GLP-1 receptor agonists, or other metabolic agents in particular patient subgroups. Targeting maladaptive adipokine signaling can also improve renal protection and potentially delay the progression of DKD beyond glucose control alone.

From a translational perspective, the integration of multi-omics platforms, organoid systems, and spatial profiling will likely expedite the validation of actionable adipokine targets. These efforts are consistent with the overarching aims of precision medicine, which seeks to convert mechanistic understanding into personalized care and sustainable health outcomes for the kidneys.

In summary, by linking molecular mechanisms with patient-centered strategies, adipokine research not only offers profound scientific insight but also holds substantial clinical utility. The detailed mechanistic and network-oriented understanding of lipid-regulating adipokines presented here provides a practical framework for early detection, precision-guided intervention, and prolonged renal protection in DKD. This approach marks a paradigm shift from targeting isolated molecules to modulating the entire network for enduring renal health. Ongoing integration of mechanistic insights with clinical innovations is crucial to fully capitalize on the potential of this approach.

Declarations

Ethics approval and consent to participate

Formatted: Shadow

iThenticate

iThenticate

Not applicable.

Consent for publication

Not applicable.

Availability of data and materials

Not applicable.

Competing interests

The authors declare that they have no competing interests.

Funding

This research received no external funding.

Authors' contributions

KY performed the literature search, investigation, original draft writing, and visualization. YF and JH contributed to literature search, investigation, and manuscript review & editing. JL contributed to conceptualization, manuscript review & 17 editing, supervision, and project administration. All authors read and approved the final manuscript.

Acknowledgements

Not applicable.

List of Abbreviations

Abbreviation	Full Name
AMPK	AMP-activated protein kinase
Bax	Bcl-2-associated X protein
CKD	Chronic kidney disease
CMKLR1	Chemokine-like receptor 1
CRH	Corticotropin-releasing hormone
ECM	Extracellular matrix

Formatted: Shadow

iThenticate

iThenticate

Abbreviation	Full Name
eGFR	Estimated glomerular filtration rate
ER	Endoplasmic reticulum
eNAMPT	Extracellular nicotinamide phosphoribosyltransferase
FIZZ3	Found in inflammatory zone 3 (Resistin)
GEC	Glomerular endothelial cell
GLP-1RA	Glucagon-like peptide-1 receptor agonist
HMGB1	High mobility group box 1
ICAM-1	Intercellular adhesion molecule-1
IL	Interleukin
IR	Insulin resistance
IRS-1	Insulin receptor substrate-1
JAK/STAT	Janus kinase / signal transducer and activator of transcription
LCN2	Lipocalin-2
LC3	Microtubule-associated protein 1A/1B-light chain 3
MAPK	Mitogen-activated protein kinase
MCP-1	Monocyte chemoattractant protein-1
mTOR	Mechanistic target of rapamycin
NAD^+	Nicotinamide adenine dinucleotide
NLRP3	NOD-, LRR- and pyrin domain-containing protein 3
NO	Nitric oxide
NGAL	Neutrophil gelatinase-associated lipocalin
NF-κB	Nuclear factor kappa-light-chain-enhancer of activated B cells
PGC-1α	Peroxisome proliferator-activated receptor gamma coactivator 1-alpha
PI3K	Phosphoinositide 3-kinase
PTECs	Proximal tubular epithelial cells
RAS	Renin-angiotensin system
ROS	Reactive oxygen species
SIRT1	Sirtuin 1
Smad	Mothers against decapentaplegic homolog
SGLT2i	Sodium-glucose cotransporter 2 inhibitor
Th	T helper cell
TNF-α	Tumor necrosis factor-alpha
TGF-β	Transforming growth factor-beta
TLR4	Toll-like receptor 4
VEGF	Vascular endothelial growth factor

Note: Abbreviations are defined at first use in the text and compiled here for clarity.

Formatted: Shadow

iThenticate

iThenticate

Table 2. Mechanistic roles of adipokines in DKD

Mechanism	Sub-Category	Adipokine	EffectDirection	KeyTargets/Pathways	FunctionalOutcome	Refs
Metabolic dysregulation	Impaired insulin signaling	Resistin	Harmful	ER stress, JNK/NF-κB, ↑IRS-1 Ser307, ↓Akt/eNOS	Promotes insulin resistance	[49]
		Chemerin	Harmful	p38 MAPK, NF-κB, ↓IRS-1/Akt	Reduces glucose uptake, insulin resistance	[50–51]
		Leptin	Harmful	↑TGF-β1, ↑VEGF	Glomerular hypertrophy, fibrosis	[52]
		Adiponectin	Protective	AMPK, PI3K/Akt	Enhances insulin signaling, reduces oxidative stress /inflammation	[53]
		Leptin/Adiponectin ratio	Diagnostic	-	Reflects metabolic–inflammatory status	[54]
	Lipid overload	Visfatin	Harmful	↑CD36, NF-κB, MCP-1, TGF-β1	Lipid accumulation, fibrosis	[55–56]
		Chemerin	Harmful	↑CD36, NF-κB	Lipotoxicity, mitochondrial dysfunction	[55]
	Energy sensing	Adiponectin	Protective	AMPK, mTOR inhibition	↑FA β-oxidation, ↓ROS	[57–58]
		Visfatin	Harmful	↓SIRT1	Impaired mitophagy, energy imbalance	[59]
Inflammation & immune dysregulation	Immune recruitment/polarization	Chemerin	Harmful	ChemR23	Monocyte/dendritic cell chemotaxis	[60]
		Leptin	Harmful	$CD4^+$ T Th1/Th17, M1 macrophage	↑IL-6, TNF-α	[61]
		Resistin	Harmful	-	↑IL-1β, TNF-α, endothelial adhesion	[62]
	Inflammasome/pathway activation	Chemerin	Harmful	MAPK, NF-κB	Amplifies tubular inflammation	[63]
		Lipocalin-2	Harmful	HMGB1–TLR4, NLRP3	↑IL-1β/IL-18, fibrosis	[64–65]
	Protective	Adiponectin	Protective	AMPK, PPARα	↓NF-κB, TNF-α, IL-1β	[53]
		Vaspin	Protective	ERK/NF-κB suppression	↓Tubular inflammation	[66]
Oxidative stress & mitochondrial dysfunction	Oxidative stress	Adiponectin	Protective	AMPK, PGC-1α, ↓NADPH oxidase	↑Mitochondrial biogenesis, ↓ROS	[53,74]
		Leptin	Harmful	↑NADPH oxidase, PI3K/Akt, JAK/STAT	↑ROS, oxidative damage	[75–77]
		Chemerin	Harmful	CMKLR1, p38 MAPK	↑ROS, inflammation	[38,78]
		Lipocalin-2	Harmful	-	Mitochondrial dysfunction, oxidative damage	[79]
		Irisin	Protective	AMPK/SIRT1/PGC-1α	↑Mitochondrial function, ↓ROS	[80–81]
Endothelial dysfunction	Activation & abnormalities	Adiponectin	Protective	AMPK/eNOS	↑NO, ↓NF-κB, vascular protection	[86–87]
		Leptin	Harmful	JAK2/STAT3, PI3K/Akt	↑Adhesion molecules, ROS	[89–90]
		Chemerin	Harmful	ERK1/2, p38 MAPK	↑Inflammation, ROS, ↓NO	[36]
		Lipocalin-2	Harmful	↑NADPH oxidase	↑Endothelial permeability	[91–92]
		Irisin	Protective	-	Improves endothelial function	[88]
Glomerular-tubular injury & fibrosis	Fibrosis	Leptin	Harmful	PI3K, βRII	↑Collagen, fibronectin deposition	[98]
		Resistin	Harmful	TLR4/P65, RAS	↑Angiotensinogen, fibrosis	[99–100]
		Chemerin	Harmful	TGF-β1/Smad/CTGF	↑Collagen deposition	[39]
		Adiponectin	Protective	↓TGF-β, NF-κB, MCP-1	↓Fibrosis, maintains structure	[101]
		Irisin	Protective	↓TGF-β1/Smad4, β-catenin	Reverses EMT, ↓fibrosis	[102]
		Lipocalin-2	Harmful	-	↑ROS, apoptosis	[79]
Apoptosis–autophagy imbalance	Apoptosis & autophagy	Adiponectin	Protective	AMPK/mTOR	↑Autophagy, ↓ROS-induced apoptosis	[53]
		Leptin	Harmful	JAK/STAT, PI3K/Akt	↑Podocyte apoptosis, mesangial hypertrophy	[106]
		Lipocalin-2	Harmful	↑mTOR, DRP1	↑Apoptosis, tubular atrophy	[79]

Note: FA, fatty acid; EMT, epithelial–mesenchymal transition; ROS, reactive oxygen species; RAS, renin–angiotensin system; NO, nitric oxide; ECM, extracellular matrix; mtDAMPs, mitochondrial damage-associated molecular patterns; mTOR, mechanistic target of rapamycin

Formatted: Shadow

iThenticate

iThenticate

References

1. Hoogeveen EK. The epidemiology of diabetic kidney disease. Kidney Dial. 2022;2(3):433-42. https://doi.org/10.3390/kidneydial2030038
2. Li J, Guo K, Qiu J, Xue S, Pi L, Li X, Huang G, Xie Z, Zhou Z. Epide miological status, development trends, and risk factors of disability-adjusted life years due to diabetic kidney disease: a systematic analysis of Global Burden of Disease Study 2021. Chin Med J (Engl). 2025;138(5):568-78. https://doi.org/10.1097/CM9.0000000000003428
3. He Y, Wang X, Li L, Liu M, Wu Y, Chen R, He J, Mai W, Li X. Globa l, regional, and national prevalence of chronic type 2 diabetic kidney disea se from 1990 to 2021: a trend and health inequality analyses based on the Global Burden of Disease Study 2021. J Diabetes. 2025;17(5):e70098. https://doi.org/10.1111/1753-0407.70098
4. Tuttle KR, Agarwal R, Alpers CE, Bakris GL, Brosius FC, Kolkhof P, Uri barri J. Molecular mechanisms and therapeutic targets for diabetic kidney d isease. Kidney Int. 2022;102(2):248-60. https://doi.org/10.1016/j.kint.2022.05.012
5. Pestel J, Blangero F, Watson J, Pirola L, Eljaafari A. Adipokines in obesit y and metabolic-related-diseases. Biochimie. 2023;212:48-59. https://doi.org/10.1016/j.biochi.2023.04.008
6. Hemat Jouy S, Mohan S, Scichilone G, Mostafa A, Mahmoud AM. Adipo kines in the crosstalk between adipose tissues and other organs: implication

Formatted: Shadow

iThenticate

iThenticate

s in cardiometabolic diseases. Biomedicines. 2024;12(9):2129. https://doi.org/10.3390/biomedicines12092129

7. Kautzky-Willer A, Harreiter J, Pacini G. Sex and gender differences in risk, pathophysiology and complications of type 2 diabetes mellitus. Endocr Rev. 2016;37(3):278-316. https://doi.org/10.1210/er.2015-1137
8. Carrero JJ. Gender differences in chronic kidney disease: underpinnings and therapeutic implications. Nat Rev Nephrol. 2010;6(5):271-80. https://doi.org/10.1038/nrneph.2010.36
9. Morselli E, Santos RS, Criollo A, Nelson MD, Palmer BF. The impact of oestrogen and progesterone on kidney disease. Nat Rev Nephrol. 2022;18(5):321-35. https://doi.org/10.1038/s41581-022-00538-3
10. Saxton SN, Clark BJ, Withers SB, Eringa EC, Heagerty AM. Mechanistic links between obesity, diabetes, and blood pressure: role of perivascular adipose tissue. Physiol Rev. 2019;99(4):1701-63. https://doi.org/10.1152/physrev.00034.2018
11. Kita S, Maeda N, Shimomura I. Interorgan communication by exosomes, adipose tissue, and adiponectin in metabolic syndrome. J Clin Invest. 2019;129(10):4041-9. https://doi.org/10.1172/JCI129193
12. Fujishima Y, Maeda N, Matsuda K, Masuda S, Mori T, Fukuda S, Sekimoto R, Yamaoka M, Obata Y, Kita S, Nishizawa H, Funahashi T, Ranscht B, Shimomura I. Adiponectin association with T-cadherin protects against neointima proliferation and atherosclerosis. FASEB J. 2017;31(4):1571-83. htt

Formatted: Shadow

iThenticate

iThenticate

ps://doi.org/10.1096/fj.201601064R

13. Obata Y, Kita S, Koyama Y, Fukuda S, Takeda H, Takahashi M, Fujishima Y, Nagao H, Masuda S, Tanaka Y, Nakamura Y, Nishizawa H, Funahashi T, Ranscht B, Izumi Y, Bamba T, Fukusaki E, Hanayama R, Shimada S, Maeda N, Shimomura I. Adiponectin/T-cadherin system enhances exosome biogenesis and decreases cellular ceramides by exosomal release. JCI Insight. 2018;3(8):e99680. https://doi.org/10.1172/jci.insight.99680
14. Chaurasia B, Tippetts TS, Mayoral Monibas R, Liu J, Li Y, Wang L, Wilkerson JL, Sweeney CR, Pereira RF, Sumida DH, Maschek JA, Cox JE, Kaddai V, Lancaster GI, Siddique MM, Poss A, Pearson M, Satapati S, Zhou H, McLaren DG, Previs SF, Chen Y, Qian Y, Petrov A, Wu M, Shen X, Yao J, Nunes CN, Howard AD, Wang L, Erion MD, Rutter J, Holland WL, Kelley DE, Summers SA. Targeting a ceramide double bond improves insulin resistance and hepatic steatosis. Science. 2019;365(6451):386-92. https://doi.org/10.1126/science.aav3722
15. Luo J, He Z, Li Q, Lv M, Cai Y, Ke W, Niu X, Zhang Z. Adipokines in atherosclerosis: unraveling complex roles. Front Cardiovasc Med. 2023;10:1235953. https://doi.org/10.3389/fcvm.2023.1235953
16. Obradovic M, Sudar-Milovanovic E, Soskic S, Essack M, Arya S, Stewart AJ, Gojobori T, Isenovic ER. Leptin and obesity: role and clinical implication. Front Endocrinol (Lausanne). 2021;12:585887. https://doi.org/10.3389/fendo.2021.585887

Formatted: Shadow

iThenticate

iThenticate

17. Minokoshi Y, Kim YB, Peroni OD, Fryer LG, Müller C, Carling D, Kahn BB. Leptin stimulates fatty-acid oxidation by activating AMP-activated protein kinase. Nature. 2002;415(6869):339-43. https://doi.org/10.1038/415339a

18. Mantzoros CS, Magkos F, Brinkoetter M, Sienkiewicz E, Dardeno TA, Kim SY, Hamnvik OP, Koniaris A. Leptin in human physiology and pathophysiology. Am J Physiol Endocrinol Metab. 2011;301(4):E567-84. https://doi.org/10.1152/ajpendo.00315.2011

19. Tripathi D, Kant S, Pandey S, Ehtesham NZ. Resistin in metabolism, inflammation, and disease. FEBS J. 2020;287(15):3141-9. https://doi.org/10.1111/febs.15322

20. Qatanani M, Szwergold NR, Greaves DR, Ahima RS, Lazar MA. Macrophage-derived human resistin exacerbates adipose tissue inflammation and insulin resistance in mice. J Clin Invest. 2009;119(3):531-9. https://doi.org/10.1172/JCI37273

21. Gao R, Wu Y, Yang Q, Chen L, Chen J, Wang B, Liu Z, Jin J, Li J, Wu G. The interaction of apelin and FGFR1 ameliorated the kidney fibrosis through suppression of TGFβ-induced endothelial-to-mesenchymal transition. Oxid Med Cell Longev. 2023;2023:5012474. https://doi.org/10.1155/2023/5012474

22. Hu G, Wang Z, Zhang R, Sun W, Chen X. The role of apelin/apelin receptor in energy metabolism and water homeostasis: a comprehensive narrative review. Front Physiol. 2021;12:632886. https://doi.org/10.3389/fphys.2021.

Formatted: Shadow

iThenticate

iThenticate

632886

23. Gourdy P, Cazals L, Thalamas C, Sommet A, Calvas F, Galitzky M, Vinel C, Dray C, Hanaire H, Castan-Laurell I, Valet P. Apelin administration improves insulin sensitivity in overweight men during hyperinsulinaemic-euglycaemic clamp. Diabetes Obes Metab. 2018;20(1):157-64. https://doi.org/10.1111/dom.13055
24. Fukuhara A, Matsuda M, Nishizawa M, Segawa K, Tanaka M, Kishimoto K, Matsuki Y, Murakami M, Ichisaka T, Murakami H, Watanabe E, Takagi T, Akiyoshi M, Ohtsubo T, Kihara S, Yamashita S, Makishima M, Funahashi T, Yamanaka S, Hiramatsu R, Matsuzawa Y, Shimomura I. Visfatin: a protein secreted by visceral fat that mimics the effects of insulin. Science. 2005;307(5708):426-30. https://doi.org/10.1126/science.1097243
25. Revollo JR, Körner A, Mills KF, Satoh A, Wang T, Garten A, Dasgupta B, Sasaki Y, Wolberger C, Townsend RR, Milbrandt J, Kiess W, Imai S. Nampt/PBEF/Visfatin regulates insulin secretion in beta cells as a systemic NAD biosynthetic enzyme. Cell Metab. 2007;6(5):363-75. https://doi.org/10.1016/j.cmet.2007.09.003
26. Garten A, Petzold S, Körner A, Imai S, Kiess W. Nampt: linking NAD biology, metabolism and cancer. Trends Endocrinol Metab. 2009;20(3):130-8. https://doi.org/10.1016/j.tem.2008.10.004
27. Zhu Y, Xu P, Huang X, Shuai W, Liu L, Zhang S, Zhao R, Hu X, Wang G. From rate-limiting enzyme to therapeutic target: the promise of NAMP

Formatted: Shadow

iThenticate

iThenticate

T in neurodegenerative diseases. Front Pharmacol. 2022;13:920113. https://doi.org/10.3389/fphar.2022.920113

28. Rodriguez M, Xu H, Hernandez A, Ingraham J, Canizales J, Arce FT, Camp SM, Briggs S, Ooi A, Burke JM, Song JH, Garcia JGN. NEDD4 E3 ligase-catalyzed NAMPT ubiquitination and autophagy activation are essential for pyroptosis-independent NAMPT secretion in human monocytes. Cell Commun Signal. 2025;23(1):157. https://doi.org/10.1186/s12964-025-02164-5
29. Camp SM, Ceco E, Evenoski CL, Danilov SM, Zhou T, Chiang ET, Moreno-Vinasco L, Mapes B, Zhao J, Gursoy G, Brown ME, Adyshev DM, Siddiqui SS, Quijada H, Sammani S, Letsiou E, Saadat L, Yousef M, Wang T, Liang J, Garcia JG. Unique Toll-like receptor 4 activation by NAMPT/PBEF induces NFκB signaling and inflammatory lung injury. Sci Rep. 2015;5:13135. https://doi.org/10.1038/srep13135
30. Chen Y, Liang Y, Hu T, Wei R, Cai C, Wang P, Wang L, Qiao W, Feng L. Endogenous Nampt upregulation is associated with diabetic nephropathy inflammatory-fibrosis through the NF-κB p65 and Sirt1 pathway; NMN alleviates diabetic nephropathy inflammatory-fibrosis by inhibiting endogenous Nampt. Exp Ther Med. 2017;14(5):4181-93. https://doi.org/10.3892/etm.2017.5098
31. Mageswari R, Sridhar MG, Nandeesha H, Parameshwaran S, Vinod KV. Irisin and visfatin predicts severity of diabetic nephropathy. Indian J Clin Biochem. 2019;34(3):342-6. https://doi.org/10.1007/s12291-018-0749-7

Formatted: Shadow

iThenticate

iThenticate

32. Hida K, Wada J, Eguchi J, Zhang H, Baba M, Seida A, Hashimoto I, Okada T, Yasuhara A, Nakatsuka A, Shikata K, Hourai S, Futami J, Watanabe E, Matsuki Y, Hiramatsu R, Akagi S, Makino H, Kanwar YS. Visceral adipose tissue-derived serine protease inhibitor: a unique insulin-sensitizing adipocytokine in obesity. Proc Natl Acad Sci U S A. 2005;102(30):10610-5. https://doi.org/10.1073/pnas.0504703102

33. Weiner J, Zieger K, Pippel J, Heiker JT. Molecular mechanisms of vaspin action - from adipose tissue to skin and bone, from blood vessels to the brain. Adv Exp Med Biol. 2019;1111:159-88. https://doi.org/10.1007/5584_2018_241

34. Jung HN, Jung CH. The role of anti-inflammatory adipokines in cardiometabolic disorders: moving beyond adiponectin. Int J Mol Sci. 2021;22(24):13529. https://doi.org/10.3390/ijms222413529

35. Nakatsuka A, Yamaguchi S, Eguchi J, Kakuta S, Iwakura Y, Sugiyama H, Wada J. A vaspin-HSPA1L complex protects proximal tubular cells from organelle stress in diabetic kidney disease. Commun Biol. 2021;4(1):373. https://doi.org/10.1038/s42003-021-01902-y

36. Xie Y, Liu L. Role of chemerin/ChemR23 axis as an emerging therapeutic perspective on obesity-related vascular dysfunction. J Transl Med. 2022;20(1):141. https://doi.org/10.1186/s12967-021-03220-7

37. Zhao L, Leung LL, Morser J. Chemerin forms: their generation and activity. Biomedicines. 2022;10(8):2018. https://doi.org/10.3390/biomedicines100820

Formatted: Shadow

iThenticate

iThenticate

18

38. Shang J, Wang L, Zhang Y, Zhang S, Ning L, Zhao J, Cheng G, Liu D, Xiao J, Zhao Z. Chemerin/ChemR23 axis promotes inflammation of glomer ular endothelial cells in diabetic nephropathy. J Cell Mol Med. 2019;23(5): 3417-28. https://doi.org/10.1111/jcmm.14237
39. Wang W, Guo J, Wang D. Promotion of chemerin in rat diabetic kidney d isease through enhancement of TGF-β1/Smads/CTGF pathway. Am J Transl Res. 2021;13(9):10206-17. https://doi.org/10.1186/s12964-025-02164-5
40. Imiela AM, Stępnicki J, Zawadzka PS, Bursa A, Pruszczyk P. Chemerin as a driver of cardiovascular diseases: new perspectives and future directions. Biomedicines. 2025;13(6):1481. https://doi.org/10.3390/biomedicines1306148 1
41. Colaianni G, Cinti S, Colucci S, Grano M. Irisin and musculoskeletal healt h. Ann N Y Acad Sci. 2017;1402(1):5-9. https://doi.org/10.1111/nyas.13345
42. Qiongyue Z, Xin Y, Meng P, Sulin M, Yanlin W, Xinyi L, Xuemin S. Po st-treatment with irisin attenuates acute kidney injury in sepsis mice throug h anti-ferroptosis via the SIRT1/Nrf2 pathway. Front Pharmacol. 2022;13:85 7067. https://doi.org/10.3389/fphar.2022.857067
43. Wang PW, Pang Q, Zhou T, Song XY, Pan YJ, Jia LP, Zhang AH. Irisin alleviates vascular calcification by inhibiting VSMC osteoblastic transformat ion and mitochondria dysfunction via AMPK/Drp1 signaling pathway in ch ronic kidney disease. Atherosclerosis. 2022;346:36-45. https://doi.org/10.1016

Formatted: Shadow

iThenticate

iThenticate

/j.atherosclerosis.2022.02.007

44. Jaberi SA, Cohen A, D'Souza C, Abdulrazzaq YM, Ojha S, Bastaki S, Adeghate EA. Lipocalin-2: structure, function, distribution and role in metabolic disorders. Biomed Pharmacother. 2021;142:112002. https://doi.org/10.1016/j.biopha.2021.112002
45. Lindstrom E, Deis J, Bernlohr DA, Chen X. Lipocalin 2 in obesity and diabetes: insights into its role in energy metabolism. Endocrines. 2025;6(1):4. https://doi.org/10.3390/endocrines6010004
46. Bolignano D, Donato V, Coppolino G, Campo S, Buemi A, Lacquaniti A, Buemi M. Neutrophil gelatinase-associated lipocalin (NGAL) as a marker of kidney damage. Am J Kidney Dis. 2008;52(3):595-605. https://doi.org/10.1053/j.ajkd.2008.01.020
47. Liu X, Zhao X, Duan X, Wang X, Wang T, Feng S, Zhang H, Chen C, Li G. Knockout of NGAL aggravates tubulointerstitial injury in a mouse model of diabetic nephropathy by enhancing oxidative stress and fibrosis. Exp Ther Med. 2021;21(4):321. https://doi.org/10.3892/etm.2021.9752
48. Jiang S, Li H, Zhang L, Liu Y, Wang J, Chen Q, Zhou W, Yang H, Zhang Y, Li X, Wang S, Sun T, Liu J, Zhao K. Generic diagramming platform (GDP): a comprehensive database of high-quality biomedical graphics. Nucleic Acids Res. 2025;53(D1):D1670-6. https://doi.org/10.1093/nar/gkae973
49. Luo J, Huang L, Wang A, Liu Y, Cai R, Li W, Zhou MS. Resistin-induced endoplasmic reticulum stress contributes to the impairment of insulin sig

Formatted: Shadow

iThenticate

iThenticate

naling in endothelium. Front Pharmacol. 2018;9:1226. https://doi.org/10.3389/fphar.2018.01226

50. Zhao L, Zhou J, Abbasi F, Fathzadeh M, Knowles JW, Leung LLK, Morser J. Chemerin in participants with or without insulin resistance and diabetes. Biomedicines. 2024;12(4):924. https://doi.org/10.3390/biomedicines12040924

51. Sell H, Laurencikiene J, Taube A, Eckardt K, Cramer A, Horrighs A, Arner P, Eckel J. Chemerin is a novel adipocyte-derived factor inducing insulin resistance in primary human skeletal muscle cells. Diabetes. 2009;58(12):2731-40. https://doi.org/10.2337/db09-0277

52. Leung JC, Chan LY, Tang SC, Chu KM, Lai KN. Leptin induces TGF-beta synthesis through functional leptin receptor expressed by human peritoneal mesothelial cell. Kidney Int. 2006;69(11):2078-86. https://doi.org/10.1038/sj.ki.5000409

53. Kim Y, Park CW. Mechanisms of adiponectin action: implication of adiponectin receptor agonism in diabetic kidney disease. Int J Mol Sci. 2019;20(7):1782. https://doi.org/10.3390/ijms20071782

54. Park HS, Park SH, Seong Y, Kim HJ, Choi HY, Rhee Y, Park HC, Jhee JH. Adiponectin-to-leptin ratio and incident chronic kidney disease: sex and body composition-dependent association. J Cachexia Sarcopenia Muscle. 2024;15(4):1298-308. https://doi.org/10.1002/jcsm.13475

55. Ahmed N, Dalmasso C, Turner MB, Arthur G, Cincinelli C, Loria AS. Fr

Formatted: Shadow

iThenticate

iThenticate

om fat to filter: the effect of adipose tissue-derived signals on kidney function. Nat Rev Nephrol. 2025;21(6):417-34. https://doi.org/10.1038/s41581-025-00950-5

56. Mahmood N, Junejo AM, Jamal Q, Awan R. Association of visfatin with chronic kidney disease in a cohort of patients with and without diabetes. J Pak Med Assoc. 2010;60(11):922-6. https://doi.org/10.1016/j.biochi.2023.04.008

57. Przybyciński J, Dziedziejko V, Puchałowicz K, Domański L, Pawlik A. Adiponectin in chronic kidney disease. Int J Mol Sci. 2020;21(24):9375. https://doi.org/10.3390/ijms21249375

58. Sharma K, Ramachandrarao S, Qiu G, Usui HK, Zhu Y, Dunn SR, Ouedraogo R, Hough K, McCue P, Chan L, Falkner B, Goldstein BJ. Adiponectin regulates albuminuria and podocyte function in mice. J Clin Invest. 2008;118(5):1645-56. https://doi.org/10.1172/JCI32691

59. Morevati M, Fang EF, Mace ML, Kanbay M, Gravesen E, Nordholm A, Egstrand S, Hornum M. Roles of NAD+ in acute and chronic kidney diseases. Int J Mol Sci. 2022;24(1):137. https://doi.org/10.3390/ijms24010137

60. Wang X, Guo J, Wu Q, Niu C, Cheng G, Liu D, Liu Z, Zhao Z, Xiao J. Chemerin/chemR23 association with endothelial-mesenchymal transition in diabetic nephropathy. Int J Clin Exp Pathol. 2017;10(7):7408-16. https://doi.org/10.1186/s12964-025-02164-5

61. Kiernan K, Nichols AG, Alwarawrah Y, MacIver NJ. Effects of T cell lept

Formatted: Shadow

iThenticate

iThenticate

in signaling on systemic glucose tolerance and T cell responses in obesity. PLoS One. 2023;18(6):e0286470. https://doi.org/10.1371/journal.pone.0286470

62. Li Y, Yang Q, Cai D, Guo H, Fang J, Cui H, Gou L, Deng J, Wang Z, Zuo Z. Resistin, a novel host defense peptide of innate immunity. Front Immunol. 2021;12:699807. https://doi.org/10.3389/fimmu.2021.699807

63. Zhang H, Mu J, Du J, Feng Y, Xu W, Bai M, Zhang H. Alpha-lipoic acid could attenuate the effect of chemerin-induced diabetic nephropathy progression. Iran J Basic Med Sci. 2021;24(8):1107-16. https://doi.org/10.22038/ijbms.2021.50792.11570

64. Song E, Jahng JW, Chong LP, Sung HK, Han M, Luo C, Wu D, Boo S, Hinz B, Cooper MA, Robertson AA, Berger T, Mak TW, George I, Schulze PC, Wang Y, Xu A, Sweeney G. Lipocalin-2 induces NLRP3 inflammasome activation via HMGB1 induced TLR4 signaling in heart tissue of mice under pressure overload challenge. Am J Transl Res. 2017;9(6):2723-35. https://doi.org/10.1186/s12964-025-02164-5

65. Wu M, Yang Z, Zhang C, Shi Y, Han W, Song S, Mu L, Du C, Shi Y. Inhibition of NLRP3 inflammasome ameliorates podocyte damage by suppressing lipid accumulation in diabetic nephropathy. Metabolism. 2021;118:154748. https://doi.org/10.1016/j.metabol.2021.154748

66. Zhang GZ, Zhang K, Yang SQ, Zhang Z, Chen S, Hou BJ, Yuan JY. VASPIN reduces inflammation and endoplasmic reticulum stress of renal tubul

Formatted: Shadow

iThenticate

iThenticate

ar epithelial cells by inhibiting HMGB1 and relieves renal ischemia-reperfusion injury. Eur Rev Med Pharmacol Sci. 2020;24(17):8968-77. https://doi.org/10.26355/eurrev_202009_22839

67. Shang D, Zhao S. Molecular mechanisms of obesity predisposes to atopic dermatitis. Front Immunol. 2024;15:1473105. https://doi.org/10.3389/fimmu.2024.1473105
68. Acosta-Martinez M, Cabail MZ. The PI3K/Akt pathway in meta-inflammation. Int J Mol Sci. 2022;23(23):15330. https://doi.org/10.3390/ijms232315330
69. Su S, Ma Z, Wu H, Xu Z, Yi H. Oxidative stress as a culprit in diabetic kidney disease. Life Sci. 2023;322:121661. https://doi.org/10.1016/j.lfs.2023.121661
70. Ma X, Ma J, Leng T, Yuan Z, Hu T, Liu Q, Shen T. Advances in oxidative stress in pathogenesis of diabetic kidney disease and efficacy of TCM intervention. Ren Fail. 2023;45(1):2146512. https://doi.org/10.1080/0886022X.2022.2146512
71. Flemming N, Pernoud L, Forbes J, Gallo L. Mitochondrial dysfunction in individuals with diabetic kidney disease: a systematic review. Cells. 2022;11(16):2481. https://doi.org/10.3390/cells11162481
72. Takasu M, Kishi S, Nagasu H, Kidokoro K, Brooks CR, Kashihara N. The role of mitochondria in diabetic kidney disease and potential therapeutic targets. Kidney Int Rep. 2024;10(2):328-42. https://doi.org/10.1016/j.ekir.2024.10.035

Formatted: Shadow

iThenticate

iThenticate

73. Stanigut AM, Tuta L, Pana C, Alexandrescu L, Suceveanu A, Blebea NM, Vacaroiu IA. Autophagy and mitophagy in diabetic kidney disease-a literature review. Int J Mol Sci. 2025;26(2):806. https://doi.org/10.3390/ijms26020806

74. Zhou M, Xu A, Tam PK, Lam KS, Huang B, Liang Y, Lee IK, Wu D, Wang Y. Upregulation of UCP2 by adiponectin: the involvement of mitochondrial superoxide and hnRNP K. PLoS One. 2012;7(2):e32349. https://doi.org/10.1371/journal.pone.0032349

75. Blanca AJ, Ruiz-Armenta MV, Zambrano S, Salsoso R, Miguel-Carrasco J L, Fortuño A, Revilla E, Mate A, Vázquez CM. Leptin induces oxidative stress through activation of NADPH oxidase in renal tubular cells: antioxidant effect of L-carnitine. J Cell Biochem. 2016;117(10):2281-8. https://doi.org/10.1002/jcb.25526

76. Korczynska J, Czumaj A, Chmielewski M, Swierczynski J, Sledzinski T. The causes and potential injurious effects of elevated serum leptin levels in chronic kidney disease patients. Int J Mol Sci. 2021;22(9):4685. https://doi.org/10.3390/ijms22094685

77. Kim K, Lee EY. Excessively enlarged mitochondria in the kidneys of diabetic nephropathy. Antioxidants (Basel). 2021;10(5):741. https://doi.org/10.3390/antiox10050741

78. Xie Q, Deng Y, Huang C, Liu P, Yang Y, Shen W, Gao P. Chemerin-induced mitochondrial dysfunction in skeletal muscle. J Cell Mol Med. 2015;1

Formatted: Shadow

iThenticate

iThenticate

9(5):986-95. https://doi.org/10.1111/jcmm.12487

79. Marques E, Alves Teixeira M, Nguyen C, Terzi F, Gallazzini M. Lipocalin -2 induces mitochondrial dysfunction in renal tubular cells via mTOR path way activation. Cell Rep. 2023;42(9):113032. https://doi.org/10.1016/j.celrep. 2023.113032

80. Formigari GP, Dátilo MN, Vareda B, Bonfante ILP, Cavaglieri CR, Lopes de Faria JM, Lopes de Faria JB. Renal protection induced by physical exe rcise may be mediated by the irisin/AMPK axis in diabetic nephropathy. S ci Rep. 2022;12(1):9062. https://doi.org/10.1038/s41598-022-13054-y

81. Yano N, Zhang L, Wei D, Dubielecka PM, Wei L, Zhuang S, Zhu P, Qin G, Liu PY, Chin YE, Zhao TC. Irisin counteracts high glucose and fatty acid-induced cytotoxicity by preserving the AMPK-insulin receptor signalin g axis in C2C12 myoblasts. Am J Physiol Endocrinol Metab. 2020;318(5): E791-805. https://doi.org/10.1152/ajpendo.00219.2019

82. Masenga SK, Kabwe LS, Chakulya M, Kirabo A. Mechanisms of oxidativ e stress in metabolic syndrome. Int J Mol Sci. 2023;24(9):7898. https://doi. org/10.3390/ijms24097898

83. Xue C, Chen K, Gao Z, Bao T, Dong L, Zhao L, Tong X, Li X. Commo n mechanisms underlying diabetic vascular complications: focus on the inte raction of metabolic disorders, immuno-inflammation, and endothelial dysfu nction. Cell Commun Signal. 2023;21(1):298. https://doi.org/10.1186/s12964-022-01016-w

Formatted: Shadow

iThenticate

iThenticate

84. Yang J, Liu Z. Mechanistic pathogenesis of endothelial dysfunction in diabetic nephropathy and retinopathy. Front Endocrinol (Lausanne). 2022;13:816400. https://doi.org/10.3389/fendo.2022.816400

85. Hang X, Ma J, Wei Y, Wang Y, Zang X, Xie P, Zhang L, Zhao L. Renal microcirculation and mechanisms in diabetic kidney disease. Front Endocrinol (Lausanne). 2025;16:1580608. https://doi.org/10.3389/fendo.2025.1580608

86. Rodríguez C, Muñoz M, Contreras C, Prieto D. AMPK, metabolism, and vascular function. FEBS J. 2021;288(12):3746-71. https://doi.org/10.1111/febs.15863

87. Fawaz S, Martin Alonso A, Qiu Y, Ramnath R, Stowell-Connolly H, Gamez M, May C, Down C, Coward RJ, Butler MJ, Welsh GI, Satchell SC, Foster RR. Adiponectin reduces glomerular endothelial glycocalyx disruption and restores glomerular barrier function in a mouse model of type 2 diabetes. Diabetes. 2024;73(6):964-76. https://doi.org/10.2337/db23-0455

88. Lu J, Xiang G, Liu M, Mei W, Xiang L, Dong J. Irisin protects against endothelial injury and ameliorates atherosclerosis in apolipoprotein E-Null diabetic mice. Atherosclerosis. 2015;243(2):438-48. https://doi.org/10.1016/j.atherosclerosis.2015.10.020

89. Yan Y, Wang L, Zhong N, Wen D, Liu L. Multifaced roles of adipokines in endothelial cell function. Front Endocrinol (Lausanne). 2024;15:1490143. https://doi.org/10.3389/fendo.2024.1490143

90. Juan CC, Chuang TY, Lien CC, Lin YJ, Huang SW, Kwok CF, Ho LT. L

Formatted: Shadow

iThenticate

iThenticate

eptin increases endothelin type A receptor levels in vascular smooth muscl e cells. Am J Physiol Endocrinol Metab. 2008;294(3):E481-7. https://doi.org/10.1152/ajpendo.00103.2007

91. Liu JT, Song E, Xu A, Berger T, Mak TW, Tse HF, Law IK, Huang B, Liang Y, Vanhoutte PM, Wang Y. Lipocalin-2 deficiency prevents endotheli al dysfunction associated with dietary obesity: role of cytochrome P450 2C inhibition. Br J Pharmacol. 2012;165(2):520-31. https://doi.org/10.1111/j.1476-5381.2011.01587.x
92. Zeng XF, Lu DX, Li JM, Tan Y, Li Z, Zhou L, Xi ZQ, Zhang SM, Duan W. Performance of urinary neutrophil gelatinase-associated lipocalin, clust erin, and cystatin C in predicting diabetic kidney disease and diabetic micr oalbuminuria: a consecutive cohort study. BMC Nephrol. 2017;18(1):233. https://doi.org/10.1186/s12882-017-0620-8
93. Kleibert M, Tkacz K, Winiarska K, Małyszko J, Cudnoch-Jędrzejewska A. The role of hypoxia-inducible factors 1 and 2 in the pathogenesis of diabe tic kidney disease. J Nephrol. 2025;38(1):37-47. https://doi.org/10.1007/s40620-024-02152-x
94. Wu T, Ding L, Andoh V, Zhang J, Chen L. The mechanism of hyperglyce mia-induced renal cell injury in diabetic nephropathy disease: an update. Li fe (Basel). 2023;13(2):539. https://doi.org/10.3390/life13020539
95. Chang J, Yan J, Li X, Liu N, Zheng R, Zhong Y. Update on the mechani sms of tubular cell injury in diabetic kidney disease. Front Med (Lausann

Formatted: Shadow

iThenticate

iThenticate

e). 2021;8:661076. https://doi.org/10.3389/fmed.2021.661076

96. Kim S, Kang SW, Joo J, Han SH, Shin H, Nam BY, Park J, Yoo TH, Kim G, Lee P, Park JT. Characterization of ferroptosis in kidney tubular cell death under diabetic conditions. Cell Death Dis. 2021;12(2):160. https://doi.org/10.1038/s41419-021-03452-x

97. Baumann B, Hayashida T, Liang X, Schnaper HW. Hypoxia-inducible factor-1α promotes glomerulosclerosis and regulates COL1A2 expression through interactions with Smad3. Kidney Int. 2016;90(4):797-808. https://doi.org/10.1016/j.kint.2016.05.026

98. Han DC, Isono M, Chen S, Casaretto A, Hong SW, Wolf G, Ziyadeh FN. Leptin stimulates type I collagen production in db/db mesangial cells: glucose uptake and TGF-beta type II receptor expression. Kidney Int. 2001;59(4):1315-23. https://doi.org/10.1046/j.1523-1755.2001.0590041315.x

99. Jiang Y, Lu L, Hu Y, Li Q, An C, Yu X, Shu L, Chen A, Niu C, Zhou L, Yang Z. Resistin induces hypertension and insulin resistance in mice via a TLR4-dependent pathway. Sci Rep. 2016;6:22193. https://doi.org/10.1038/srep22193

100. Xu Z, Li W, Han J, Zou C, Huang W, Yu W, Shan X, Lum H, Li X, Liang G. Angiotensin II induces kidney inflammatory injury and fibrosis through binding to myeloid differentiation protein-2 (MD2). Sci Rep. 2021;11(1):15820. https://doi.org/10.1038/s41598-021-94987-8

101. Zhao D, Zhu X, Jiang L, Huang X, Zhang Y, Wei X, Zhao X, Du Y. Ad

Formatted: Shadow

iThenticate

iThenticate

vances in understanding the role of adiponectin in renal fibrosis. Nephrolo gy (Carlton). 2021;26(2):197-203. https://doi.org/10.1111/nep.13808

102. Yang Z, Wei J, Wang Y, Du Y, Song S, Li J, Su Z, Shi Y, Wu H. Irisin ameliorates renal tubulointerstitial fibrosis by regulating the Smad4/β-caten in pathway in diabetic mice. Diabetes Metab Syndr Obes. 2023;16:1577-93. https://doi.org/10.2147/DMSO.S407734

103. Allen DA, Harwood S, Varagunam M, Raftery MJ, Yaqoob MM. High glu cose-induced oxidative stress causes apoptosis in proximal tubular epithelial cells and is mediated by multiple caspases. FASEB J. 2003;17(8):908-10. https://doi.org/10.1096/fj.02-0130fje

104. Lindblom R, Higgins G, Coughlan M, de Haan JB. Targeting mitochondria and reactive oxygen species-driven pathogenesis in diabetic nephropathy. Rev Diabet Stud. 2015;12(1-2):134-56. https://doi.org/10.1900/RDS.2015.12.1 34

105. Xu Y, Liu L, Xin W, Zhao X, Chen L, Zhen J, Wan Q. The renoprotecti ve role of autophagy activation in proximal tubular epithelial cells in diabe tic nephropathy. J Diabetes Complications. 2015;29(8):976-83. https://doi.org /10.1016/j.jdiacomp.2015.07.021

106. Shih YL, Shih CC, Chen SY, Chen JY. Elevated serum leptin levels are a ssociated with lower renal function among middle-aged and elderly adults i n Taiwan, a community-based, cross-sectional study. Front Endocrinol (Lau sanne). 2022;13:1047731. https://doi.org/10.3389/fendo.2022.1047731

Formatted: Shadow

iThenticate

iThenticate

107. Kacso AC, Bondor CI, Coman AL, Potra AR, Georgescu CE. Determinants of visfatin in type 2 diabetes patients with diabetic kidney disease: relationship to inflammation, adiposity and undercarboxylated osteocalcin. Scand J Clin Lab Invest. 2016;76(3):217-25. https://doi.org/10.3109/00365513.2015.1137349

108. Kang YS, Lee MH, Song HK, Kim JE, Ghee JY, Cha JJ, Lee JE, Kim HW, Han JY, Cha DR. Chronic administration of visfatin ameliorated diabetic nephropathy in type 2 diabetic mice. Kidney Blood Press Res. 2016;41(3):311-24. https://doi.org/10.1159/000443433

109. Scilletta S, Leggio S, Di Marco M, Miano N, Musmeci M, Marrano N, Natalicchio A, Giorgino F, Bosco G, Di Giacomo Barbagallo F, Scamporrino A, Di Mauro S, Filippello A, Scicali R, Russello M, Spadaro L, Purrello F, Piro S, Di Pino A. Acute hyperbilirubinemia determines an early subclinical renal damage: evaluation of tubular biomarkers in cholemic nephropathy. Liver Int. 2024;44(9):2341-50. https://doi.org/10.1111/liv.16005

110. Fang F, Liu L, Yang Y, Tamaki Z, Wei J, Marangoni RG, Bhattacharyya S, Summer RS, Ye B, Varga J. The adipokine adiponectin has potent anti-fibrotic effects mediated via adenosine monophosphate-activated protein kinase: novel target for fibrosis therapy. Arthritis Res Ther. 2012;14(5):R229. https://doi.org/10.1186/ar4070

111. Perri A, Vizza D, Lupinacci S, Toteda G, De Amicis F, Leone F, Gigliotti P, Lofaro D, La Russa A, Bonofiglio R. Adiponectin secreted by tubular

Formatted: Shadow

iThenticate

iThenticate

renal cells during LPS exposure worsens the cellular inflammatory damage. J Nephrol. 2016;29(2):185-94. https://doi.org/10.1007/s40620-015-0220-2

112. Ghosh AR, Bhattacharya R, Bhattacharya S, Nargis T, Rahaman O, Duttagupta P, Raychaudhuri D, Liu CS, Roy S, Ghosh P, Khanna S, Chaudhuri T, Tantia O, Haak S, Bandyopadhyay S, Mukhopadhyay S, Chakrabarti P, Ganguly D. Adipose recruitment and activation of plasmacytoid dendritic cells fuel metaflammation. Diabetes. 2016;65(11):3440-52. https://doi.org/10.2337/db16-0331

113. Raschke S, Elsen M, Gassenhuber H, Sommerfeld M, Schwahn U, Brockmann B, Jung R, Wisløff U, Tjønna AE, Raastad T, Hallén J, Norheim F, Drevon CA, Romacho T, Eckardt K, Eckel J. Evidence against a beneficial effect of irisin in humans. PLoS One. 2013;8(9):e73680. https://doi.org/10.1371/journal.pone.0073680

114. Jodeiri Farshbaf M, Alviña K. Multiple roles in neuroprotection for the exercise derived myokine irisin. Front Aging Neurosci. 2021;13:649929. https://doi.org/10.3389/fnagi.2021.649929

115. Kiguchi N, Maeda T, Kobayashi Y, Fukazawa Y, Kishioka S. Leptin enhances CC-chemokine ligand expression in cultured murine macrophage. Biochem Biophys Res Commun. 2009;384(3):311-5. https://doi.org/10.1016/j.bbrc.2009.04.121

116. Buechler C, Feder S, Haberl EM, Aslanidis C. Chemerin isoforms and activity in obesity. Int J Mol Sci. 2019;20(5):1128. https://doi.org/10.3390/ijms2

Formatted: Shadow

iThenticate

iThenticate

0051128

117. Li R, Dong F, Zhang L, Ni X, Lin G. Role of adipocytokines in endometrial cancer progression. Front Pharmacol. 2022;13:1090227. https://doi.org/10.3389/fphar.2022.1090227

118. Behnoush AH, Shobeiri P, Bahiraie P, Amirkhani N, Khalaji A, Peiman S. Chemerin levels in chronic kidney disease: a systematic review and meta-analysis. Front Endocrinol (Lausanne). 2023;14:1120774. https://doi.org/10.3389/fendo.2023.1120774

119. Yuan F, Liu YH, Liu FY, Peng YM, Tian JW. Intraperitoneal administration of the globular adiponectin gene ameliorates diabetic nephropathy in Wistar rats. Mol Med Rep. 2014;9(6):2293-300. https://doi.org/10.3892/mmr.2014.2133

120. Lai W, Luo D, Li Y, Li Y, Wang Q, Hu Z, Ye Z, Peng H. Irisin ameliorates diabetic kidney disease by restoring autophagy in podocytes. FASEB J. 2023;37(10):e23175. https://doi.org/10.1096/fj.202300420R

121. Simental-Mendía LE, Sánchez-García A, Linden-Torres E, Simental-Mendía M. Impact of glucagon-like peptide-1 receptor agonists on adiponectin concentrations: a meta-analysis of randomized controlled trials. Br J Clin Pharmacol. 2021;87(11):4140-9. https://doi.org/10.1111/bcp.14855

122. Simental-Mendía LE, Sánchez-García A, Linden-Torres E, Simental-Mendía M. Effect of glucagon-like peptide-1 receptor agonists on circulating levels of leptin and resistin: a meta-analysis of randomized controlled trials. Di

Formatted: Shadow

iThenticate

iThenticate

abetes Res Clin Pract. 2021;177:108899. https://doi.org/10.1016/j.diabres.202 1.108899

123. Wang D, Liu J, Zhong L, Li S, Zhou L, Zhang Q, Li M, Xiao X. The e ffect of sodium-glucose cotransporter 2 inhibitors on biomarkers of inflam mation: a systematic review and meta-analysis of randomized controlled tri als. Front Pharmacol. 2022;13:1045235. https://doi.org/10.3389/fphar.2022.10 45235

124. Kelly KR, Navaneethan SD, Solomon TP, Haus JM, Cook M, Barkoukis H, Kirwan JP. Lifestyle-induced decrease in fat mass improves adiponectin secretion in obese adults. Med Sci Sports Exerc. 2014;46(5):920-6. https:// doi.org/10.1249/MSS.0000000000000200

125. Jiménez-Martínez P, Ramirez-Campillo R, Alix-Fages C, Gene-Morales J, G arcía-Ramos A, Colado JC. Chronic resistance training effects on serum ad ipokines in type 2 diabetes mellitus: a systematic review. Healthcare (Base l). 2023;11(4):594. https://doi.org/10.3390/healthcare11040594

126. Koelman L, Egea Rodrigues C, Aleksandrova K. Effects of dietary patterns on biomarkers of inflammation and immune responses: a systematic revie w and meta-analysis of randomized controlled trials. Adv Nutr. 2022;13(1): 101-15. https://doi.org/10.1093/advances/nmab086

Formatted: Shadow

iThenticate

972568568971395072.docx

ORIGINALITY REPORT

10%	8%	8%	2%
SIMILARITY INDEX	INTERNET SOURCES	PUBLICATIONS	STUDENT PAPERS

PRIMARY SOURCES

1	www.frontiersin.org<br>Internet Source	2%
2	www.mdpi.com<br>Internet Source	1%
3	www.researchsquare.com<br>Internet Source	<1%
4	pmc.ncbi.nlm.nih.gov<br>Internet Source	<1%
5	e-space.mmu.ac.uk<br>Internet Source	<1%
6	lirias.kuleuven.be<br>Internet Source	<1%
7	Systems Biology of Free Radicals and Antioxidants, 2014.<br>Publication	<1%
8	Min Li, Luying Yang, Ting Li, Yanmei Miao, Jun Yang, Shaolin Chen, Xinglong Ma, Peng Xie. "Leptin's potential mechanisms in ICU-	<1%

acquired weakness", Lipids in Health and Disease, 2025
Publication

9	cyberleninka.org<br>Internet Source	<1 %
10	journalallergy.com<br>Internet Source	<1 %
11	journals.sagepub.com<br>Internet Source	<1 %
12	www.science.gov<br>Internet Source	<1 %
13	d-nb.info<br>Internet Source	<1 %
14	Submitted to University of Birmingham<br>Student Paper	<1 %
15	Anqi Tang, Yu Zhang, Ling Wu, Yong Lin, Lizeyu Lv, Liangbin Zhao, Bojun Xu, Youqun Huang, Mingquan Li. "Klotho's impact on diabetic nephropathy and its emerging connection to diabetic retinopathy", Frontiers in Endocrinology, 2023<br>Publication	<1 %
16	Submitted to Monash University<br>Student Paper	<1 %
17	repository.publisso.de<br>Internet Source	

<1 %

18 "Biomarkers in Diabetes", Springer Science and Business Media LLC, 2023
Publication
<1 %

19 Brijesh Sutariya, Madhusudan Saraf. "Betanin, isolated from fruits of Opuntia elatior Mill attenuates renal fibrosis in diabetic rats through regulating oxidative stress and TGF-β pathway", Journal of Ethnopharmacology, 2017
Publication
<1 %

20 Feiyi Duan, Jiaoyan Wu, Jiayi Chang, Haoyuan Peng et al. "Deciphering endocrine function of adipose tissue and its significant influences in obesity-related diseases caused by its dysfunction", Differentiation, 2025
Publication
<1 %

21 Ioanna Gianopoulos, Christos S Mantzoros, Stella S Daskalopoulou. "Adiponectin and Adiponectin Receptors in Atherosclerosis", Endocrine Reviews, 2024
Publication
<1 %

22 Qi Jia, Yeling Ouyang, Yiyi Yang, Shanglong Yao, Xiangdong Chen, Zhiqiang Hu. "Adipokines in pulmonary hypertension: angels or demons?", Heliyon, 2023
Publication
<1 %

23	Wanning Wang, Weixia Sun, Yanli Cheng, Zhonggao Xu, Lu Cai. "Role of sirtuin-1 in diabetic nephropathy", Journal of Molecular Medicine, 2019<br>Publication	<1 %
24	www.spandidos-publications.com<br>Internet Source	<1 %
25	Fanghong Li, Zhi Ma, Yajie Cai, Jingwei Zhou, Runping Liu. "Optimizing diabetic kidney disease animal models: Insights from a meta-analytic approach", Animal Models and Experimental Medicine, 2023<br>Publication	<1 %
26	ora.ox.ac.uk<br>Internet Source	<1 %
27	Sensen Su, Zhanchuan Ma, Hao Wu, Zhonggao Xu, Huanfa Yi. "Oxidative stress as a culprit in diabetic kidney disease", Life Sciences, 2023<br>Publication	<1 %
28	journals.lww.com<br>Internet Source	<1 %
29	link.springer.com<br>Internet Source	<1 %
30	nbscience.com<br>Internet Source	<1 %

31	tessera.spandidos-publications.com<br>Internet Source	<1 %
32	"Translational Mitochondrial Medicine", Springer Science and Business Media LLC, 2024<br>Publication	<1 %
33	Bin Wang, Chen Zhao, Yuanxin Wang, Xin Tian et al. " Exercise ameliorating myocardial injury in type 2 diabetic rats by inhibiting excessive mitochondrial fission involving increased irisin expression and phosphorylation ", Journal of Diabetes, 2023<br>Publication	<1 %
34	Dalamaga, Maria. "Interplay of adipokines and myokines in cancer pathophysiology: Emerging therapeutic implications", World Journal of Experimental Medicine, 2013.<br>Publication	<1 %
35	Maoying Wei, Xingxing Liu, Zhijuan Tan, Xiaochan Tian, Mingdi Li, Junping Wei. "Ferroptosis: a new strategy for Chinese herbal medicine treatment of diabetic nephropathy", Frontiers in Endocrinology, 2023<br>Publication	<1 %

36	Wilson José S. Pedro, Flávio V. Barbosa Júnior, Fernanda N. B. R. Alves, Lenita V. Braga et al. "Role of Adipokines Chemerin, Visfatin, and Omentin in Obesity and Their Inflammatory and Metabolic Implications", Biomedicines, 2025<br>Publication	<1 %
37	Zhi Peng, Hui Wang, Jiaoyun Zheng, Jie Wang, Yang Xiang, Chi Liu, Ming Ji, Huijun Liu, Lang Pan, Xiaoqun Qin, Xiangping Qu. "Is the proximal tubule the focus of tubulointerstitial fibrosis?", Heliyon, 2023<br>Publication	<1 %
38	assets.researchsquare.com<br>Internet Source	<1 %
39	download.bibis.ir<br>Internet Source	<1 %
40	mdpi-res.com<br>Internet Source	<1 %
41	www.tara.tcd.ie<br>Internet Source	<1 %
42	Hongdian Li, Ao Dong, Cong Liu, Pengfei He, Yu Ma, Shu Chen, Shaoning Dong, Sai Zhang, Minying Zhang, Mianzhi Zhang. "Schisandra chinensis Mixture Attenuates Diabetic Kidney Disease via VDAC1/Grp75/IP3R-Mediated	<1 %

MAMs Stabilization and Apoptosis-Autophagy Regulation", Phytomedicine, 2025
Publication

43	Komal Thapa, Neha Kanojia, Heena Khan, Amarjot Kaur, Thakur Gurjeet Singh. "Angiopoietins: A potential therapeutic target in the treatment of diabetic nephropathy", Obesity Medicine, 2025<br>Publication	<1 %
44	Stefano Gianoli, Justin Tang, Kirsten C. Odegard, Koichi Yuki, Sophia Koutsogiannaki. "Harnessing adiponectin for sepsis: current knowledge, clinical insights and future therapies", Critical Care, 2025<br>Publication	<1 %
45	cloneresources.com<br>Internet Source	<1 %
46	www.ncbi.nlm.nih.gov<br>Internet Source	<1 %
47	www.researchgate.net<br>Internet Source	<1 %
48	"Renal Fibrosis: Mechanisms and Therapies", Springer Science and Business Media LLC, 2019<br>Publication	<1 %

49 Afiat Berbudi, Shafia Khairani, Adi Tjahjadi. "Interplay Between Insulin Resistance and Immune Dysregulation in Type 2 Diabetes Mellitus: Implications for Therapeutic Interventions", ImmunoTargets and Therapy, 2025 <1%
Publication

50 Kimio Watanabe, Emiko Sato, Eikan Mishima, Mariko Miyazaki, Tetsuhiro Tanaka. "What's New in the Molecular Mechanisms of Diabetic Kidney Disease: Recent Advances", International Journal of Molecular Sciences, 2022 <1%
Publication

51 Marta Ruiz-Ortega, Monica Ruperez, Vanesa Esteban, Jesús Egido. "Molecular mechanisms of angiotensin II-induced vascular injury", Current Hypertension Reports, 2003 <1%
Publication

52 The Stress Response of Critical Illness Metabolic and Hormonal Aspects, 2016. <1%
Publication

53 Xuanke Liu, Chunjiang Zhang, Yanjie Fu, Linlin Xie, Yijing Kong, Xiaoping Yang. "Inflammation, Apoptosis, and Fibrosis in Diabetic Nephropathy: Molecular Crosstalk in Proximal Tubular Epithelial Cells and <1%

Therapeutic Implications", Current Issues in Molecular Biology, 2025
Publication

54 Kajetan Kiełbowski, Estera Bakinowska, Piotr Ostrowski, Bartłomiej Pala et al. "The Role of Adipokines in the Pathogenesis of Psoriasis", International Journal of Molecular Sciences, 2023
Publication
<1 %

55 Lili Qu, Baihai Jiao. "The Interplay between Immune and Metabolic Pathways in Kidney Disease", Cells, 2023
Publication
<1 %

56 Manupati Srikanth, Mahaboobkhan Rasool. "Resistin – A Plausible Therapeutic Target in the Pathogenesis of Psoriasis", Immunological Investigations, 2023
Publication
<1 %

57 Ron Stout, Daniel Reichert, Rebecca Kelly. "Lifestyle Medicine and the Primary Care Provider - A Practical Guide to Enabling Whole Person Care", CRC Press, 2025
Publication
<1 %

58 Sayantap Datta, Saisudha Koka, Krishna M. Boini. "Understanding the Role of Adipokines in Cardiometabolic Dysfunction: A Review of Current Knowledge", Biomolecules, 2025
Publication
<1 %

59	Shaghayegh Hemat Jouy, Sukrutha Mohan, Giorgia Scichilone, Amro Mostafa, Abeer M. Mahmoud. "Adipokines in the Crosstalk between Adipose Tissues and Other Organs: Implications in Cardiometabolic Diseases", Biomedicines, 2024<br>Publication	<1 %
60	Yucan Guan, Xianping Wei, Jicui Li, Yuexin Zhu, Ping Luo, Manyu Luo. "Obesity-related glomerulopathy: recent advances in inflammatory mechanisms and related treatments", Journal of Leukocyte Biology, 2024<br>Publication	<1 %
61	Yuya Wen, Chenling Zhao, Jie Chen, Liwei Tian, Bojin Wu, Wenting Xie, Ting Dong. "Gandouling Regulates Ferroptosis and Improves Neuroinflammation in Wilson's Disease Through the LCN2/NLRP3 Signaling Pathway", Journal of Inflammation Research, 2024<br>Publication	<1 %

Exclude quotes	Off	Exclude matches	Off
Exclude bibliography	On		
