# Supplementary material for: Adipokine networks in diabetic kidney disease: mechanistic insights and therapeutic implications
Source: Lipids Health Dis. 2026 Jan 10;25:43. doi: 10.1186/s12944-025-02851-9 (PMC12879388; doi:10.1186/s12944-025-02851-9)
Supplement: Supplementary file 3 — Supplementary Material 3. [file 12944_2025_2851_MOESM3_ESM.pdf]

105 Xingang West Road
Haizhu District,Guangzhou,China
BioGDP.com

Confirmation of Publication and Licensing Rights

BioGDP Account ID: 1431846702@qq.com
Agreement number: GDP2025I2HEB2

To whom this may concern,

This document is to confirm that **Ke Yang** from **Dongzhimen Hospital, Beijing University of Chinese Medicine, Beijing, China** has been granted a license to use the BioGDP content, including graphics,templates and other original artwork, appearing in the attached completed graphic pursuant to BioGDP's Academic License Terms.This license permits BioGDP content to be sublicensed for use in **Lipids in Health and Disease** publications.

All rights and ownership of BioGDP content are reserved by BioGDP. All completed graphics must be accompanied by the following citation:“Created with biogdp.com”.

BioGDP content included in the completed graphic is not licensed for any commercial uses beyond publication in a journal.For any commercial use of this figure,users may contact BioGDP Support at gdp-coloring@outlook.com.

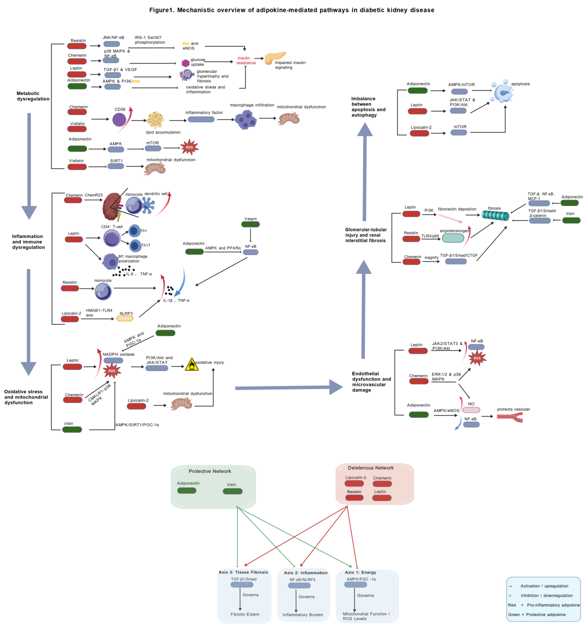

For any questions regarding this document,or other questions about publishing with BioGDP please contact BioGDP Support at gdp-coloring@outlook.com.
