# Supplementary material for: Adipokine networks in diabetic kidney disease: mechanistic insights and therapeutic implications
Source: Lipids Health Dis. 2026 Jan 10;25:43. doi: 10.1186/s12944-025-02851-9 (PMC12879388; doi:10.1186/s12944-025-02851-9)
Supplement: Supplementary file 4 — Supplementary Material 4. [file 12944_2025_2851_MOESM4_ESM.docx]

Supplementary Table 2. Mechanistic roles of adipokines in DKD

| **1.Metabolic dysregulation** | | | | | |
| --- | --- | --- | --- | --- | --- |
| **Sub-Category** | **Adipokine** | **EffectDirection** | **KeyTargets/Pathways** | **FunctionalOutcome** | **Refs** |
| Impaired insulin signaling | Resistin | Harmful | ER stress, JNK/NF-κB, ↑IRS-1 Ser307, ↓Akt/eNOS | Promotes insulin resistance | [49] |
|  | Chemerin | Harmful | p38 MAPK, NF-κB, ↓IRS-1/Akt | Reduces glucose uptake, insulin resistance | [50–51] |
|  | Leptin | Harmful | ↑TGF-β1, ↑VEGF | Glomerular hypertrophy, fibrosis | [52] |
|  | Adiponectin | Protective | AMPK, PI3K/Akt | Enhances insulin signaling, reduces oxidative stress/inflammation | [53] |
|  | Leptin/Adiponectin ratio | Diagnostic | - | Reflects metabolic–inflammatory status | [54] |
| Lipid overload | Visfatin | Harmful | ↑CD36, NF-κB, MCP-1, TGF-β1 | Lipid accumulation, fibrosis | [55–56] |
|  | Chemerin | Harmful | ↑CD36, NF-κB | Lipotoxicity, mitochondrial dysfunction | [55] |
| Energy sensing | Adiponectin | Protective | AMPK, mTOR inhibition | ↑FA β-oxidation, ↓ROS | [57–58] |
|  | Visfatin | Harmful | ↓SIRT1 | Impaired mitophagy, energy imbalance | [59] |
|  | | | | | |
| **2.Inflammation & immune dysregulation** | | | | | |
| **Sub-Category** | **Adipokine** | **EffectDirection** | **KeyTargets/Pathways** | **FunctionalOutcome** | **Refs** |
| Immune recruitment/polarization | Chemerin | Harmful | ChemR23 | Monocyte/dendritic cell chemotaxis | [60] |
|  | Leptin | Harmful | CD4⁺ T Th1/Th17, M1 macrophage | ↑IL-6, TNF-α | [61] |
|  | Resistin | Harmful | - | ↑IL-1β, TNF-α, endothelial adhesion | [62] |
| Inflammasome/pathway activation | Chemerin | Harmful | MAPK, NF-κB | Amplifies tubular inflammation | [63] |
|  | Lipocalin-2 | Harmful | HMGB1–TLR4, NLRP3 | ↑IL-1β/IL-18, fibrosis | [64–65] |
| Anti-inflammatory | Adiponectin | Protective | AMPK, PPARα | ↓NF-κB, TNF-α, IL-1β | [53] |
|  | Vaspin | Protective | ERK/NF-κB suppression | ↓Tubular inflammation | [66] |
|  | | | | | |
| **3.Oxidative stress & mitochondrial dysfunction** | | | | | |
| **Sub-Category** | **Adipokine** | **EffectDirection** | **KeyTargets/Pathways** | **FunctionalOutcome** | **Refs** |
| Oxidative stress | Adiponectin | Protective | AMPK, PGC-1α, ↓NADPH oxidase | ↑Mitochondrial biogenesis, ↓ROS | [53,74] |
|  | Leptin | Harmful | ↑NADPH oxidase, PI3K/Akt, JAK/STAT | ↑ROS, oxidative damage | [75–77] |
|  | Chemerin | Harmful | CMKLR1, p38 MAPK | ↑ROS, inflammation | [38,78] |
|  | Lipocalin-2 | Harmful | - | Mitochondrial dysfunction, oxidative damage | [79] |
|  | Irisin | Protective | AMPK/SIRT1/PGC-1α | ↑Mitochondrial function, ↓ROS | [80–81] |
|  | | | | | |
| **4. Endothelial dysfunction** | | | | | |
| **Sub-Category** | **Adipokine** | **EffectDirection** | **KeyTargets/Pathways** | **FunctionalOutcome** | **Refs** |
| Activation & abnormalities | Adiponectin | Protective | AMPK/eNOS | ↑NO, ↓NF-κB, vascular protection | [86–87] |
|  | Leptin | Harmful | JAK2/STAT3, PI3K/Akt | ↑Adhesion molecules, ROS | [89–90] |
|  | Chemerin | Harmful | ERK1/2, p38 MAPK | ↑Inflammation, ROS, ↓NO | [36] |
|  | Lipocalin-2 | Harmful | ↑NADPH oxidase | ↑Endothelial permeability | [91–92] |
|  | Irisin | Protective | - | Improves endothelial function | [88] |
|  | | | | | |
| **5.Glomerular-tubular injury & fibrosis** | | | | | |
| **Sub-Category** | **Adipokine** | **EffectDirection** | **KeyTargets/Pathways** | **FunctionalOutcome** | **Refs** |
| Fibrosis | Leptin | Harmful | PI3K, βRII | ↑Collagen, fibronectin deposition | [98] |
|  | Resistin | Harmful | TLR4/P65, RAS | ↑Angiotensinogen, fibrosis | [99–100] |
|  | Chemerin | Harmful | TGF-β1/Smad/CTGF | ↑Collagen deposition | [39] |
|  | Adiponectin | Protective | ↓TGF-β, NF-κB, MCP-1 | ↓Fibrosis, maintains structure | [101] |
|  | Irisin | Protective | ↓TGF-β1/Smad4, β-catenin | Reverses EMT, ↓fibrosis | [102] |
|  | Lipocalin-2 | Harmful | - | ↑ROS, apoptosis | [79] |
|  | | | | | |
| **6.Apoptosis–autophagy imbalance** | | | | | |
| **Sub-Category** | **Adipokine** | **EffectDirection** | **KeyTargets/Pathways** | **FunctionalOutcome** | **Refs** |
| Apoptosis & autophagy | Adiponectin | Protective | AMPK/mTOR | ↑Autophagy, ↓ROS-induced apoptosis | [53] |
|  | Leptin | Harmful | JAK/STAT, PI3K/Akt | ↑Podocyte apoptosis, mesangial hypertrophy | [106] |
|  | Lipocalin-2 | Harmful | ↑mTOR, DRP1 | ↑Apoptosis, tubular atrophy | [79] |

Note: FA, fatty acid; EMT, epithelial–mesenchymal transition; ROS, reactive oxygen species; RAS, renin–angiotensin system; NO, nitric oxide; ECM, extracellular matrix; mtDAMPs, mitochondrial damage-associated molecular patterns; mTOR, mechanistic target of rapamycin
